# Modulation of Albumin Esterase Activity by Warfarin and Diazepam

**DOI:** 10.3390/ijms252111543

**Published:** 2024-10-27

**Authors:** Daria A. Belinskaia, Anastasia A. Batalova, Polina A. Voronina, Vladimir I. Shmurak, Mikhail A. Vovk, Alexander M. Polyanichko, Tomash S. Sych, Kamila V. Samodurova, Vasilisa K. Antonova, Anastasia A. Volkova, Bogdan A. Gerda, Richard O. Jenkins, Nikolay V. Goncharov

**Affiliations:** 1 Sechenov Institute of Evolutionary Physiology and Biochemistry, Russian Academy of Sciences, pr. Torez 44, 194223 St. Petersburg, Russia; 2 St. Petersburg State University, 7/9 Universitetskaya nab., 199034 St. Petersburg, Russiaa.polyanichko@spbu.ru (A.M.P.);; 3 Leicester School of Allied Health Sciences, De Montfort University, The Gateway, Leicester LE1 9BH, UK

**Keywords:** albumin, esterases, *p*-nitrophenyl acetate, inhibitory analysis, NMR, spectrophotometry, molecular modeling

## Abstract

Data are accumulating on the hydrolytic activity of serum albumin towards esters and organophosphates. Previously, with the help of the technology of proton nuclear magnetic resonance (^1^H NMR) spectroscopy, we observed the yield of acetate in the solution of bovine serum albumin and *p*-nitrophenyl acetate (NPA). Thus, we showed that albumin possesses true esterase activity towards NPA. Then, using the methods of molecular docking and molecular dynamics, we established site Sudlow I as the catalytic center of true esterase activity of albumin. In the present work, to expand our understanding of the molecular mechanisms of albumin pseudoesterase and true esterase activity, we investigated—in experiments in vitro and in silico—the interaction of anticoagulant warfarin (WRF, specific ligand of site Sudlow I) and benzodiazepine diazepam (DIA, specific ligand of site Sudlow II) with albumins of different species, and determined how the binding of WRF and DIA affects the hydrolysis of NPA by albumin. It was found that the characteristics of the binding modes of WRF in site Sudlow I and DIA in site Sudlow II of human (HSA), bovine (BSA), and rat (RSA) albumins have species differences, which are more pronounced for site Sudlow I compared to site Sudlow II, and less pronounced between HSA and RSA compared to BSA. WRF competitively inhibits true esterase activity of site Sudlow I towards NPA and does not affect the functioning of site Sudlow II. Diazepam can slow down true esterase activity of site Sudlow I in noncompetitive manner. It was concluded that site Sudlow I is more receptive to allosteric modulation compared to site Sudlow II.

## 1. Introduction

**Figure 1:**
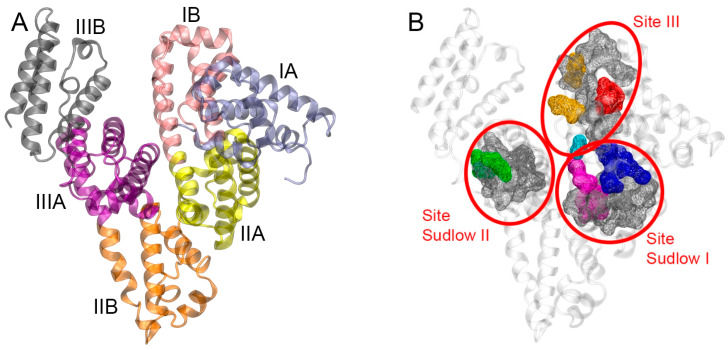
Structure of human serum albumin (HSA). (**A**) HSA domain structure; ice-blue, pink, yellow, orange, purple, and black ribbons represent domains IA, IB, IIA, IIB, IIIA, and IIIB, respectively. (**B**) Binding sites of HSA; the backbone of the protein is shown with a gray ribbon; nonpolar residues of the binding sites are shown with a gray surface; polar residues of the binding sites are shown with colored surfaces (Tyr150, His242, and Arg257, located at the bottom of site Sudlow I, are marked in blue; Lys195, Lys199, Arg218 and Arg222, located at the entrance of site Sudlow I, are marked in purple; Tyr411 and Arg410, located at the entrance of site Sudlow II, are marked in green; Tyr138 and Tyr161, located at the entrance of site III, are marked in red; Arg117 and Arg186, located at the base of site III, are marked in orange; Ser193, located at the boundary between sites I and III, is marked in cyan).

Albumin is the main protein in the blood of mammals, where its concentration is 500–700 µM [1,2]. The three-dimensional structure of the protein molecule contains three homologous domains (I, II, and III); each of them consists of two subdomains, A and B (Figure 1A) [3].

Albumin is capable of binding almost all known drugs, and many nutraceuticals and toxic compounds. Three major ligand-binding sites have been identified in albumin (site Sudlow I, site Sudlow II, and site III, Figure 1B), as well as several secondary ones [4]. Site Sudlow I is a flexible multi-compartment pocket. The site consists of a central zone connected to three separate compartments. The interior of the site is predominantly nonpolar. However, the site contains two polar regions: one at its bottom (Tyr150, His242, Arg257, marked in blue in Figure 1B), and the other at its entrance (Lys195, Lys199, Arg218, Arg222, marked in purple in Figure 1B). Site Sudlow I binds mainly bulky heterocyclic compounds with a negative charge, often delocalized on a nonpolar framework. Warfarin (WRF) is a typical ligand of Sudlow I [5]. Site Sudlow II belongs to subdomain IIIA. Based on crystallographic analysis, site Sudlow II of HSA is a nonpolar cavity with a single polar area, which includes Tyr411 and Arg410 and borders the entrance to the site (marked in green in Figure 1B). Ligands of Sudlow II typically contain a peripheral negative charge, such as ibuprofen, diazepam (DIA), and other benzodiazepines [5]. The third main binding site (site III) is located in subdomain IB and binds bilirubin, hemin, and sulfonamide. Site III includes two polar areas. The first one is positioned at the entrance of the site and consists of Tyr138 and Tyr161 (marked in red in Figure 1B). The second region at the base of the site includes Arg117 and Arg186 (in orange in Figure 1B). Polar residue Ser193 (in cyan in Figure 1B) serves as the conventional boundary between sites I and III [6].

Numerous experiments have demonstrated pseudoesterase (irreversible binding of a substrate to a protein) or true esterase (binding of a substrate to the active center of a protein followed by dissociation of the complex into the unmodified protein and the products) activity of albumin towards various esters, including organophosphates (OPs) [7]. Moreover, *p*-nitrophenyl acetate (NPA, a typical substrate for studying carboxylesterases) is commonly used to investigate the hydrolytic activity of albumin [8]. The hydrolysis product of NPA, nitrophenol, is yellow in color with an absorption peak at a wavelength of 400–412 nm and can be observed with the help of spectrophotometer. NPA shows the maximal affinity for Tyr411 of site Sudlow II, which is the main center of pseudoesterase activity in the protein [8]. Most authors connect the presence of true esterase activity in albumin with the slow deacylation of Tyr411 [7,9]. The first publication in which the existence of at least two different sites responsible for two types of activity of albumin—true and pseudoesterase—was substantiated, appeared in 1972 [10]. Later, Dr. Vilanova and colleagues showed that albumin has catalytic activity towards OPs [11,12,13].

**Scheme 1:**
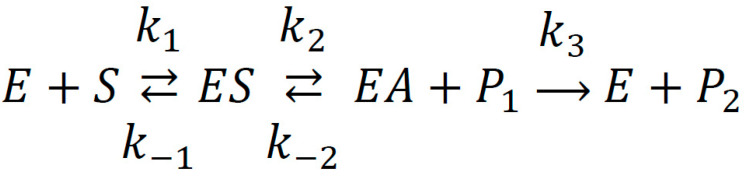
Interaction between albumin and *p*-nitrophenyl acetate (NPA). S—substrate, P1—p-nitrophenol, P2—acetate.

Based on our longstanding investigation devoted to the hydrolytic activity of albumin, we proposed a kinetic scheme for the interaction of the protein with the model substrate NPA [9]. At the first stage of the reaction, the ligand binds to site Sudlow II (ES), then the rapid release of nitrophenol (product P1) and the acetylation of Tyr411 tyrosine (EA) occur. At the second phase, NPA binds to the site of true esterase activity, where it is hydrolyzed to acetate (product P2) and *p*-nitrophenol (Scheme 1).

Previous studies on the kinetics of the interaction of albumin with substrates were based only on the detection of the yield of nitrophenol (P1 in Scheme 1), which is the product of both esterase and pseudoesterase activity of the protein. The kinetics of pseudo- and true esterase activity can be separated by observing the yield of acetate (P2 in Scheme 1). The issue is that acetate is non-chromogenic and cannot be detected using spectrophotometry. In our previous study [14], with the help of the technology of proton nuclear magnetic resonance (^1^H NMR) spectroscopy, we observed the yield of acetate during the interaction of bovine serum albumin (BSA) with NPA. Thus, we substantiated that albumin possesses true esterase activity towards NPA in a site other than the site of pseudoesterase activity of albumin. Using the methods of molecular docking and molecular dynamics, we substantiated that the catalytic tyrosine Tyr150 in site Sudlow I is responsible for true esterase activity in the protein. The next logical step in studying albumin as an enzyme is to perform an inhibitory analysis of its hydrolytic activity. The purpose of the current work is to apply in vitro and in silico methods to describe the features of binding of WRF and DIA (specific ligands of sites Sudlow I and II, respectively) to albumins of different species and to investigate the effect of WRF and DIA on the hydrolytic activity of the protein towards NPA.

## 2. Results

At the first phase of the investigation, with the help of various methodologies, we studied the binding of WRF and DIA to sites Sudlow I and II of albumins of three species as follows: human (HSA), bovine (BSA), and rat (RSA). We determined species differences in binding modes of the ligands and, for each species, described the methodological features of the analysis depending on what experimental data on the structure of the protein are currently available.

### 2.1. Interaction of WRF and DIA with Human Serum Albumin: Validation of Molecular Modeling Methods

The most reliable way to study the molecular features of the interaction of a ligand with a protein is to experimentally determine the three-dimensional structure of their complex using X-ray diffraction analysis or cryogenic electron microscopy [15,16]. If the structure of a particular protein–ligand complex has not yet been obtained, the interaction between the protein and the ligand can be confirmed by various biochemical methods in vitro (calorimetry, spectrofluorimetry, etc.), based on the fact that the structural properties of macromolecules change after the binding of ligands. Then, based on the data from the biochemical experiments, it is possible to specify the details of protein–ligand interaction using molecular modeling methods.

Crystal structures of HSA complexes with R-stereoisomer of WRF and DIA were resolved by X-ray diffraction and are available in the protein data bank (PDB), entries 2BXD [5] and 2BXF [5], respectively. We used these experimental data to validate the application of computational methods in studying the functional characteristics of albumin. For this purpose, we investigated the interaction of HSA with R-stereoisomer of WRF and DIA by molecular modeling methods and then compared the obtained result of molecular modeling with known experimental structures. Further, when we talk about WRF in computational experiments, we mean R-warfarin, while a racemate mixture of WRF is used in in vitro experiments.

**Figure 2:**
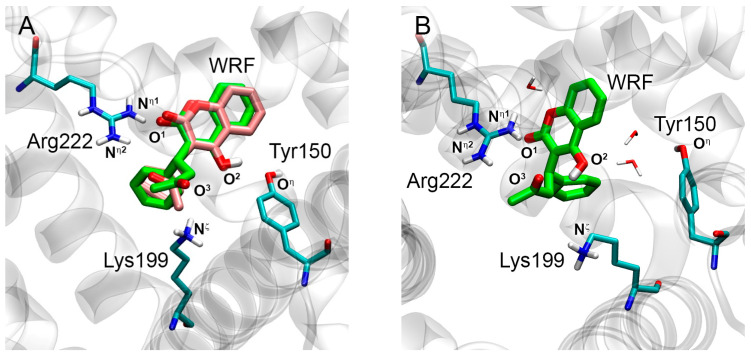
Binding modes of warfarin (WRF) in site Sudlow I of HSA obtained by molecular docking procedure (**A**) and 50 ns molecular dynamics (MD) simulation (**B**). Green shows the binding modes of WRF according to molecular modeling data, pink shows the binding mode of WRF according to X-ray diffraction analysis (PBD structures 2BXD [5]). Cyan, blue, red and white sticks represent carbon, nitrogen, oxygen and hydrogen atoms, respectively. The designations of atoms are given, the distances between which determine the pose of the ligand molecule inside the binding site. Water molecules located in the vicinity of WRF according to MD simulation are shown. Nonpolar hydrogens are omitted for clarity.

**Figure 3:**
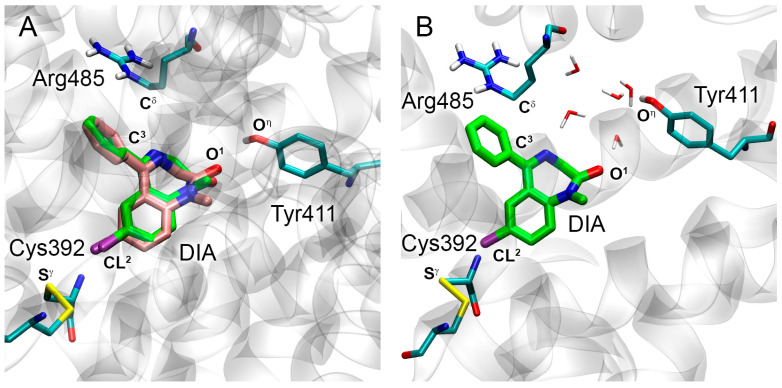
Binding modes of diazepam (DIA) in site Sudlow II of HSA obtained by molecular docking procedure (**A**) and 50 ns MD simulation (**B**). Green shows the binding modes of DIA according to molecular modeling data, pink shows the binding mode of DIA according to X-ray diffraction analysis (PBD structures 2BXF [5]). Cyan, blue, red, yellow, violet and white sticks represent carbon, nitrogen, oxygen, sulfur, chlorine and hydrogen atoms, respectively. The designations of atoms are given, the distances between which determine the pose of the ligand molecule inside the binding site. Water molecules located in the vicinity of DIA according to MD simulation are shown. Nonpolar hydrogens are omitted for clarity.

First of all, we performed the molecular docking of WRF and DIA into Sudlow sites I and II, respectively. The result is presented in Figure 2A and Figure 3A.

The key distances between functionally significant atoms according to molecular docking and X-ray analysis are presented in Table 1. The values of root mean square deviation (RMSD) between the X-ray and docked poses are 0.85 and 0.79 Å for WRF and DIA, respectively (Table 1). According to the obtained values, the result of molecular docking is in good agreement with the experimental data. The only exception is the position of the ketone group of WRF relative to the guanidine group of Arg222. According to X-ray, the ketone group interacts with Arg222, and according to molecular docking data, it is turned in the opposite direction and interacts with Lys199 (Figure 2A). The pose of DIA inside site Sudlow II obtained by molecular docking repeats the pose of the ligand obtained by X-ray (Figure 3A).

In the next step, the stability of the resulting WRF–HSA and DIA–HSA complexes was simulated by 50 ns molecular dynamics (MD). The mean and final values of the distances between functionally significant atoms were calculated from the obtained trajectories (Table 1). According to the results of MD, WRF remains bound within site Sudlow I throughout 50 ns of the simulation (Figure 2B). Although atom O^1^ of the ligand remains in the vicinity of Arg222 (Table 1), the hydrogen bond between this oxygen atom and the side chain of the arginine breaks. The hydroxyl group of WRF (atom O^2^ and the hydroxyl hydrogen) forms a hydrogen bond with the hydroxyl group of Tyr150 at the beginning of the simulation. Then, atoms O^2^ of WRF and O^η^ of Tyr150 move away from each other and the distance between them increases (Table 1). However, visual inspection shows that these groups still interact through a water molecule (Figure 2B). During the simulation, the ketone group of WRF (atom O^3^) forms a hydrogen bond with the side chain of Lys199 or with Arg222. We believe that the torsional rotation of group -C(O)-CH_3_ of WRF allows atom O^3^ to alternately interact with Lys199 (conformation according to molecular docking, shown in green in Figure 2A) and Arg222 (conformation in the crystal structure according to X-ray, shown in pink in Figure 2A).

According to MD, DIA remains bound within site Sudlow II throughout 50 ns of the simulation (Figure 3B). The chlorine atom remains bound close to sulfur atoms Sγ of residues Cys392 and Cys438. It is known that halogen substituents of low molecular weight compounds can form halogen bonds with the sulfur atoms of cysteine and methionine residues [17]. It has already been suggested that a halogen bond is formed between DIA and Cys392/438 of albumin [18]. The phenyl group of diazepam remains bound near the guanidine group of Arg485 so that the planes of these groups are parallel, that is, in this case, apparently, a π-cation interaction forms. Based on the geometry of the atoms, it can be argued that atom O^1^ of DIA forms a hydrogen bond with the hydroxyl group of Tyr411 in the crystal structure of the HSA–DIA complex (Figure 3A). During the simulation, the distance between these atoms increases (Table 1) and the bond breaks. However, visual inspection shows that these groups still interact through a water molecule (Figure 3B). Thus, in the case both of WRF and DIA, the hydrogen bonds involving oxygen and hydrogen atoms do not persist in water solution; a water molecule intervenes in the interactions. At the same time, the halogen bond, cation-π interactions, and the hydrogen bonds involving nitrogen and hydrogen atoms do persist.

To test the reproducibility of MD, we ran two more simulations for HSA–WRF and HSA–DIA complexes (i.e., the total number of runs was three for each ligand). The conformational characteristics (root mean square deviation, radius of gyration, and root mean square fluctuation calculated for the Cα-atoms of HSA) for all the runs are shown in Figure S1. According to the obtained data, the MD simulation is reproducible, so we further applied single-trajectory MD.

Thus, the result obtained by the combined use of molecular docking and MD have good reproducibility and conformity with experimental data and makes it possible to clarify the details of the HSA interaction with WRF and DIA in a water solution. It follows that the computer modeling methods used here can be applied to study the functional characteristics of albumin of other species (BSA and RSA).

### 2.2. Interaction of WRF and DIA with Bovine Serum Albumin

BSA is widely used as a model albumin in in vitro experiments due to its low cost and accessibility. However, the effectiveness of the binding of various ligands differs for BSA and HSA [19,20,21,22,23,24]. The crystal structure of ligand-free BSA was obtained in 2012 [25]. Later, the structures of BSA with 3,5-diiodosalicylic acid (ligand of both Sudlow sites [26]), naproxen (ligand of site Sudlow II [27]), and ketoprofen (ligand of site Sudlow I [28]) were obtained. However, the three-dimensional crystal structures of BSA complexes with warfarin and diazepam have not yet been resolved. In the absence of experimental data, the interaction of BSA with WRF and DIA can be studied in vitro using spectroscopic methods and in silico with the help of molecular modeling methods.

#### 2.2.1. Interaction of WRF and DIA with BSA According to Spectrofluorimetry

One of the main spectrometric methods for analyzing the binding of ligands to proteins is fluorescence spectroscopy. The method is based on the effect of a decrease in the fluorescence intensity of a protein, which can be a result of various processes in the system, such as the formation of a protein–ligand complex, in which the environment of the fluorophore changes (static quenching), and collisions of excited particles with unexcited ones (dynamic quenching). The intrinsic fluorescence emission of BSA after excitation at 280 nm is due to the presence of two tryptophan residues in the protein molecule, Trp134 and Trp213 [29]. Trp213 is a part of site Sudlow I and corresponds to Trp214 of HSA and RSA. Trp134 is unique to bovine albumin and is located on the surface of BSA molecule in subdomain IB.

**Figure 4:**
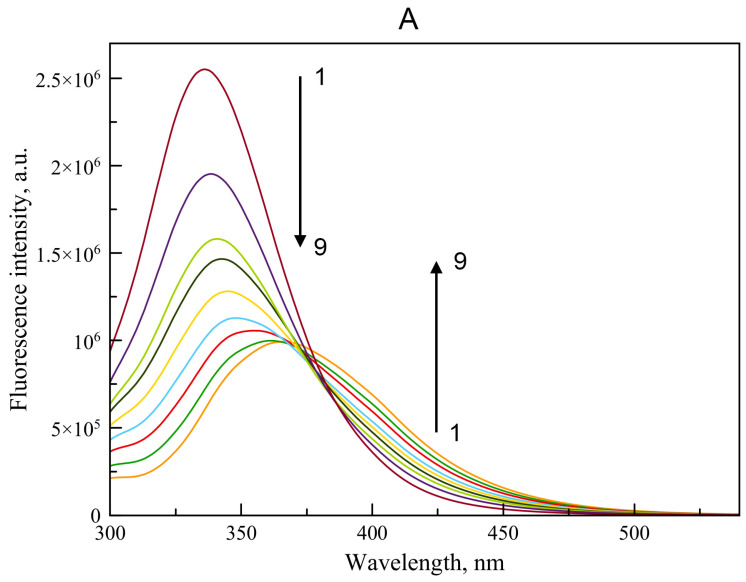

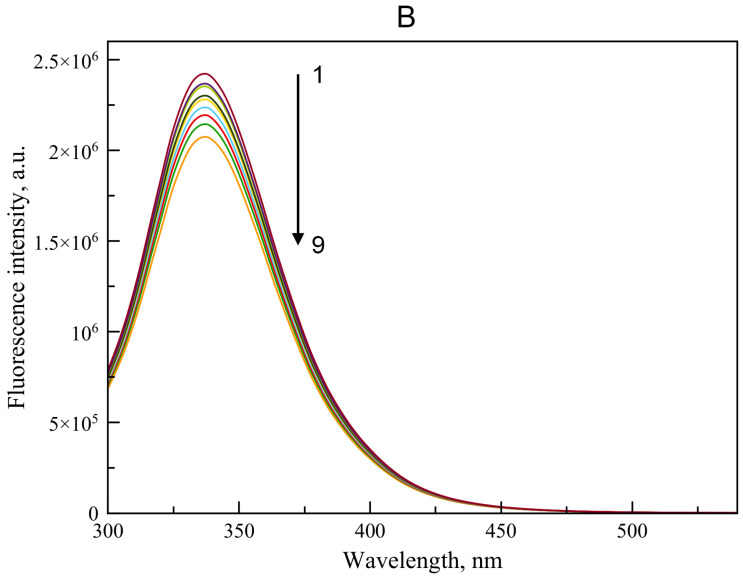
Emission spectrum of BSA when its fluorescence is quenched by WRF (**A**) and DIA (**B**). The arrow indicates the change in ligand concentration: 1 → 9 corresponds to concentrations of 0, 14.7, 24.9, 32.3, 42.0, 54.6, 71.0, 92.3, 120.0 µM. The BSA concentration was 15 μM. For each ligand concentration, the spectrum was recorded three times; the figure displays the average curves.

Using spectrofluorimetry, we analyzed the interaction of BSA with WRF and DIA. Figure 4 shows the emission spectra of BSA after its incubation with WRF (Figure 4A) and DIA (Figure 4B) at 37 °C. We chose this temperature instead of room temperature, keeping in mind our earlier in vivo experiment in rats, in which we studied modulators of albumin esterase activity as possible components of therapy for acute organophosphate poisoning [30]. After adding WRF or DIA at increasing concentrations, BSA fluorescence decreases, indicating the formation of the complexes between the ligands and the protein (Figure 4).

In the case of WRF, a quenching of intrinsic fluorescence is observed due to interaction of the ligand with the protein molecule. A similar effect was observed by Li et al. for the interaction of WRF with HSA [31]. In particular, the quenching can be caused, among other things, by energy transfer due to the partial overlap between the absorption spectra of the ligand and the emission spectra of the protein (Figure 4A). Thus, in the crystal structure of the HSA–WRF complex [5], the distance between the phenyl group of WRF and the side chain of Trp214 is about 5 Å. In the complex BSA–WRF, obtained by molecular docking (see Section 2.2.2), the distance between the aromatic ring of WRF and Trp213 is about 8 Å. Of course, besides this, the process of changing the environment of the molecule is quite probable. The absence of such an effect for DIA (Figure 4B) suggests that this ligand binds in site Sudlow II, distant from Trp213. The spectrum of similar shape was obtained by Lou et al. when studying interaction of BSA with another benzodiazepine, clonazepam [32]. However, other factors may be involved, but this requires further research, which is beyond the scope of this paper.

Thus, according to spectrofluorometric data, WRF and DIA are capable of complex formation with BSA. The energy transfer effect in the case of WRF (Figure 4A) indicates that this ligand interacts with site Sudlow I, which includes the fluorophore Trp213 (Trp214 in HSA). The absence of a similar effect for DIA (Figure 4B) suggests that this ligand binds in site Sudlow II, distant from Trp213. This result was expected since there is no reason to believe that WRF and DIA, being able to bind to HSA, do not interact with BSA. Moreover, the interaction of BSA with WRF and benzodiazepines has already been studied with the help of spectrofluorometric methods much more thoroughly than in our work [32,33]. However, we considered it necessary to evaluate the possibility of the complex formation of WRF and DIA with the specific BSA preparation that we used in our studies. It is known that commercial preparations of albumin are inhomogeneous; in different samples, the percentage of albumin with the oxidized form of Cys34 varies from 52% to 87% [34,35,36]. Albumin oxidation affects its affinity for ligands [37]. Our spectrofluorometric experiments showed that the commercial BSA that we used in further inhibitory analysis of the enzymatic activity of albumin is capable of complexation with WRF and DIA.

#### 2.2.2. Interaction of WRF and DIA with BSA According to Molecular Modeling

**Figure 5:**
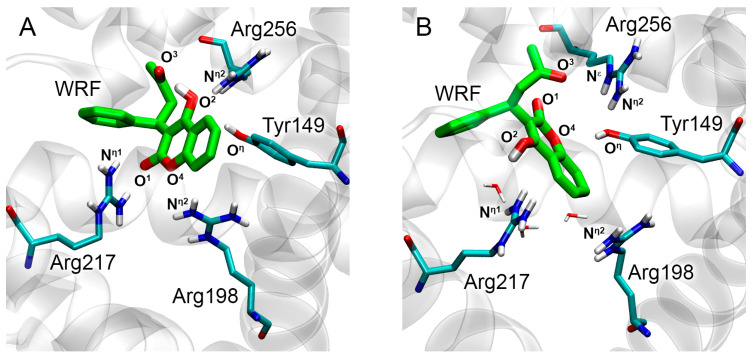
Binding modes of WRF in site Sudlow I of BSA obtained by molecular docking procedure (**A**) and 50 ns MD simulation (**B**). Cyan, blue, red and white sticks represent carbon, nitrogen, oxygen and hydrogen atoms, respectively. Carbon atoms of WRF are shown in green. The designations of atoms are given, the distances between which determine the pose of the ligand molecule inside the binding site. Water molecules located in the vicinity of WRF according to MD simulation are shown. Nonpolar hydrogens are omitted for clarity.

To determine the specific features of ligand binding to Sudlow sites of BSA at the molecular level, the interaction of BSA with WRF and DIA was studied using in silico methods. Molecular docking of WRF and DIA was performed at sites Sudlow I and II of BSA, respectively. The crystal structure of BSA (PDB entry 4JK4 [26]) was used as a three-dimensional model of the protein. The conformational behavior of the resulting complexes was simulated by the method of MD. Table 2 shows the key distances between the functionally significant atoms of WRF, DIA, and BSA according to the molecular docking procedure and MD simulation. In the BSA–WRF complex, obtained by the molecular docking procedure, the carboxyl oxygen of WRF (O^1^) binds near the side chain of Arg217; the hydroxyl oxygen (O^2^) of WRF interacts with the side chain of Arg256; the cyclic oxygen (O^4^) of WRF binds near the side chain of Arg198; and the ketone oxygen (O^3^) of the ligand forms an intramolecular hydrogen bond with the hydroxyl hydrogen (Figure 5A). During MD simulation, these contacts break; however, a stable hydrogen bond is formed between the ketone oxygen of WRF (O^3^) and the guanidine group of Arg256 (Table 2, Figure 5B). The conformation of WRF in site Sudlow I of BSA is different to the conformation of WRF in site Sudlow I of HSA, both in the initial and the end conformation of the simulation. This effect appears to be due to the fact that Lys199 and Arg222 of site Sudlow I of HSA are replaced by Arg198 and Lys221 in BSA, which affects the geometry of the site and the conformation of WRF in the site.

**Figure 6:**
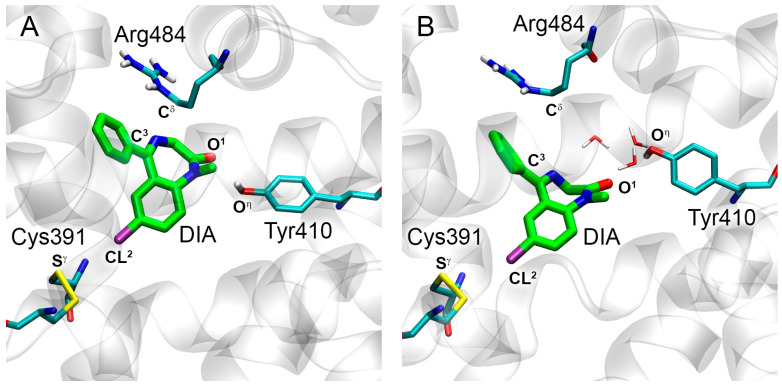
Binding modes of DIA in site Sudlow II of BSA obtained by molecular docking procedure (**A**) and 50 ns MD simulation (**B**). Cyan, blue, red, yellow, violet and white sticks represent carbon, nitrogen, oxygen, sulfur, chlorine and hydrogen atoms, respectively. Carbon atoms of DIA are shown in green. The designations of atoms are given, the distances between which determine the pose of the ligand molecule inside the binding site. Water molecules located in the vicinity of DIA according to MD simulation are shown. Nonpolar hydrogens are omitted for clarity.

The conformation of DIA in site Sudlow II of BSA (Figure 6A) is similar to the conformation of the ligand in HSA. Atom O^1^ of DIA forms a hydrogen bond with the hydroxyl group of Tyr410, the phenyl ring of the drug forms a cation-π interaction with the guanidine group of Arg484, and the chlorine atom forms a halogen bond with the sulfur atom of Cys391. The geometry of the complex of DIA with site Sudlow II of BSA persists throughout the entire simulation (Figure 6B). As in the case of HSA, the hydrogen bond between atom O^1^ and the hydroxyl group of Tyr410 exists only for a short period of the simulation (Table 2). However, when the direct hydrogen bond breaks, these groups interact through one or two water molecules (Figure 6B). The halogen bond between the chlorine atom of DIA and Cys391 of BSA and the cation-π interaction between the phenyl ring of the drug and Arg484 of BSA persists throughout the long period of the simulation.

### 2.3. Interaction of WRF and DIA with Rat Serum Albumin

*Rattus norvegicus* is one of the main models used in testing bioactive compounds in vivo [38,39,40,41]. Some xenobiotics have similar characteristics of interaction with HSA and RSA [42,43]. However, the effectiveness of the binding of some bioactive compounds differs between HSA and RSA [44,45]. As mentioned above, in our earlier in vivo experiment in rats, we studied modulators of albumin esterase activity as components of therapy for acute organophosphate poisoning [30]. That is why it is important to study the interactions of WRF and DIA with sites Sudlow of RSA. This information can help understand the extent to which the results obtained on rats can be extrapolated to human organism.

RSA is the most “problematic” albumin of the HSA–BSA–RSA trio. Due to the high cost of commercial RSA, its use in biochemical experiments is limited. The three-dimensional structure of RSA has not yet been resolved experimentally, that is why it is more difficult to run computational experiments with rat albumin compared to HSA and BSA. In the absence of experimental data, the three-dimensional structure of a protein can be constructed by homology modeling, which builds a three-dimensional model of the protein based on its primary sequence and the known three-dimensional structures of homologous proteins [46]. In the current work, to study the WRF and DIA interaction with RSA in silico, we first prepared a three-dimensional model of rat albumin using homology modeling.

#### 2.3.1. Building the Three-Dimensional Model of RSA

We have previously described the construction of the three-dimensional structure of RSA [47]. Briefly, based on the results of a search for similar three-dimensional structures in the PDB database, 14 homolog proteins were selected, which are serum albumins from various animals as follows: four structures of both HSA and BSA, three of domestic horse, two of wild rabbit, and one of domestic sheep. At the next stage, multiple alignment of the primary sequences of the selected homolog proteins was performed. Then, using the Modeller program (University of California at San Francisco, San Francisco, CA, USA) [48], 30 possible RSA structures were generated based on the selected templates. The lowest discrete optimized protein energy (DOPE) score among the resulting models was −1.78, and this structure was selected as the RSA model. According to the literature, model construction can be considered successful when the DOPE value is less than −1 [49,50]. The structure of the obtained model was optimized by the energy minimization method. The quality of the optimized model was assessed using the MolProbity service (Duke University, Durham, NC, USA) [51]. The overall quality assessment reached extremely high values; 99% of the structures from the comparative set have worse indicators than the model that we built.

Since we planned to use the homologous model for studying the interaction of RSA with WRF and DIA, the resulting structure was checked for the presence of “defects” in the Sudlow sites (amino acids with an abnormal conformation of the side chain, C^β^-atoms deviating by more than 0.25 Å from the characteristic position, bond angles and bond lengths with uncharacteristic values, amino acids in the forbidden region of the Ramachandran map) [47]. We found that among the amino acid residues of site Sudlow I, there was not a single one that belonged to the forbidden region of the Ramanchadran map, had an uncharacteristic side chain conformation, or covalent bond geometry. Also, in this region there were neither steric overlaps nor C^β^-atoms deviating from the characteristic position. The only exception was the amino acid Ala151, in which the value of angle C^α^-C-N deviated by 5° from the normal. Site Sudlow II did not contain amino acids that belong to the forbidden region of the Ramachandran map or have uncharacteristic side chain conformations, covalent bond geometry, or deviant positions of C^β^-atoms. Steric interferences were also absent [47]. The data obtained indicate that the homology-based model of RSA is of good quality; it does not experience any serious steric interference, does not have multiple violations in the conformation of amino acid residues, and is therefore suitable for further computational experiments. Alignment of the structure of RSA that we obtained by homology modeling with the RSA structure from the AlphaFold Protein Structure Database (the library of predicted protein structures [52]), performed with the help of PyMOL v2.5.3 (Schrödinger LLC, New York, NY, USA [53]), showed that root mean square deviation (RMSD) between them is equal to 0.82 Å.

#### 2.3.2. Interaction of WRF and DIA with RSA According to Molecular Modeling

**Figure 7:**
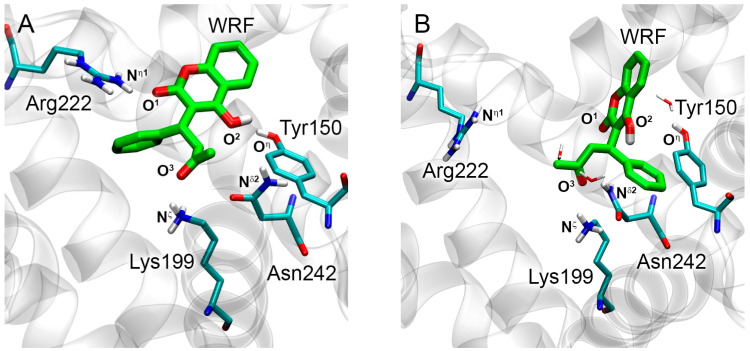
Binding modes of WRF in site Sudlow I of RSA obtained by molecular docking procedure (**A**) and 50 ns MD simulation (**B**). Cyan, blue, red and white sticks represent carbon, nitrogen, oxygen and hydrogen atoms, respectively. Carbon atoms of WRF are shown in green. The designations of atoms are given, the distances between which determine the pose of the ligand molecule inside the binding site. Water molecules located in the vicinity of WRF according to MD simulation are shown. Nonpolar hydrogens are omitted for clarity.

At the next stage, molecular docking of WRF and DIA into sites Sudlow I and II of RSA, respectively, was performed. The conformational behavior of the obtained structures was simulated by the method of MD. Table 3 shows the key distances between the functionally significant atoms of WRF, DIA, and RSA according to the molecular docking procedure and MD simulation. According to the molecular docking procedure, the binding mode of WRF in site Sudlow I of RSA is similar to the binding mode of the compound in the complex with HSA. The carboxyl oxygen of WRF (O^1^) binds near the side chain of Arg222, the hydroxyl oxygen (O^2^) interacts with the hydroxyl group of Tyr150, and the ketone oxygen (O^3^) forms a hydrogen bond with the cationic group of Lys199 (Figure 7A). Within 50 ns, contacts of WRF with Arg222 and Lys199 break (Table 3, Figure 7B). Oxygen O^3^ moves to the vicinity of Asn242 during the simulation, and a hydrogen bond is formed between them at the end of the simulation (Table 3, Figure 7B). On the contrary, in the complex of WRF with HSA, the hydrogen bond between WRF and Tyr150 of RSA is preserved during the simulation (Table 3, Figure 7B). It is interesting to note that WRF keeps a hydrogen bond with Lys199 in the complex with HSA, while it switches to Asn242 in the complex with RSA. Apparently, this is due to the fact that there is a less flexible histidine at position 242 in the HSA molecule. In general, despite the listed differences between RSA and HSA, the mechanism of binding of WRF to albumins of these species is closer to each other compared to BSA.

**Figure 8:**
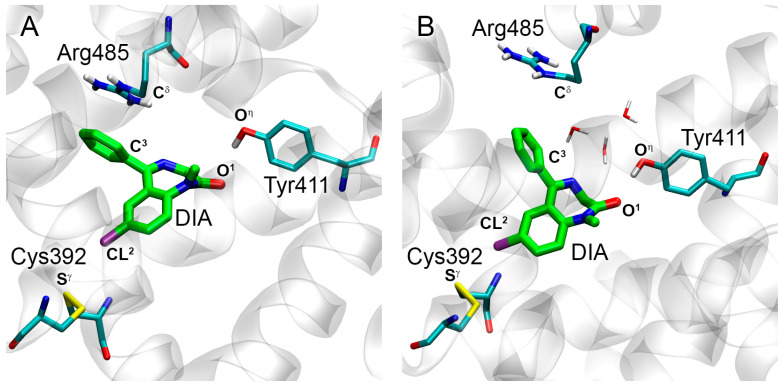
Binding modes of DIA in site Sudlow II of RSA obtained by molecular docking procedure (**A**) and 50 ns MD simulation (**B**). Cyan, blue, red, yellow, violet and white sticks represent carbon, nitrogen, oxygen, sulfur, chlorine and hydrogen atoms, respectively. Carbon atoms of DIA are shown in green. The designations of atoms are given, the distances between which determine the pose of the ligand molecule inside the binding site. Water molecules located in the vicinity of DIA according to MD simulation are shown. Nonpolar hydrogens are omitted for clarity.

The conformation of DIA in site Sudlow II of RSA (Figure 8A) is similar to the ligand conformation in the complexes with HSA and BSA; atom O^1^ of DIA forms a hydrogen bond with the hydroxyl group of Tyr411, the phenyl moiety of the drug forms a cation-π interaction with the side chain of Arg485, and the chlorine atom of DIA forms a halogen bond with the sulfur atom of Cys392. The geometry of the complex of DIA with site Sudlow II of RSA is preserved throughout the 50 ns simulation (Figure 8B). Like in HSA, the hydrogen bond between atom O^1^ and Tyr411 of RSA exists only for a short period of the simulation (the average distance between O^1^ of DIA and O^η^ of Tyr411 of RSA is 5.9 Å, Table 3). The chlorine atom maintains a halogen bond with the sulfur atom of Cys392. The phenyl group of DIA and the guanidine group of Arg485 preserve a cation-π interaction for most of the simulation. Thus, the mechanism of interaction of DIA with site Sudlow II of HSA, BSA, and RSA is almost identical and has much fewer species differences compared to the interaction of WRF with site Sudlow I.

In the next step, for the example of BSA, we studied in vitro and in silico how WRF and DIA affect the hydrolytic activity of albumin towards NPA.

### 2.4. Effect of WRF and DIA on Albumin Esterase Activity Towards NPA According to NMR Spectroscopy

**Figure 9:**
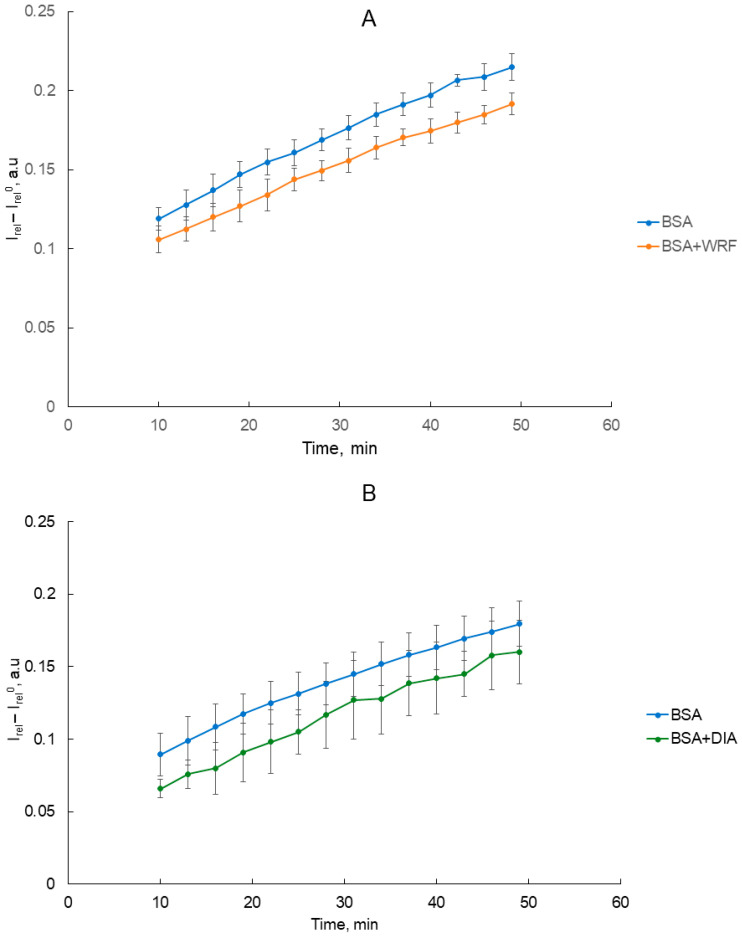
Time dependence of the relative integral intensity of the signal of acetate during the hydrolysis of NPA (3.6 mM) by BSA (180 μM) in the presence of WRF (360 μM, (**A**)) and DIA (360 μM, (**B**)). I_rel_ was calculated as the ratio of the integral signal intensity of the acetate group (1.86–1.81 ppm) to the integral signal intensity of the internal standard trimethylsilylpropanesulfonate (DSS, 0.0–−0.3 ppm, in the experiment with WRF) or dimethyl sulfoxide (DMSO, 2.65–2.55 ppm, in the experiment with DIA) [14]. I_rel_^0^ was taken as zero intensity, which is the relative integral intensity of the range 1.86–1.81 ppm (corresponding to the acetate signal) of the spectrum of pure BSA (for control measurements without an inhibitor) or the spectrum of a mixture of BSA with an inhibitor without substrate (for measurements with inhibitors).

Earlier, we applied the method of spectrophotometry to investigate the effect of CBDP and ethopropazine (inhibitors of carboxylesterase and butyrylcholinesterase, CES and BChE, respectively) on true esterase activity of BSA [54]. Here, using NMR spectroscopy, we determined how WRF and DIA affect the release of acetate during the interaction of BSA and NPA (as it is described by Scheme 1, acetate is a product of true esterase but not the pseudoesterase activity of albumin). The procedure of detection is similar to our previous experiments [14]. The time dependence of the relative integral signal intensity of the free acetate group (I_rel_, 1.86–1.81 ppm) in the absence and presence of inhibitors was plotted. Dimethyl sulfoxide (DMSO, 2.65–2.55 ppm) and sodium trimethylsilylpropanesulfonate (DSS, 0.0–−0.3 ppm) were used as the internal standard for I_rel_ calculation in the experiments with DIA and WRF, respectively. This was done for the convenience of calculations since DMSO is used as a solvent for WRF, and in the case of WRF it is more convenient to use DSS as an internal standard. The experiment was complicated by the presence of acetate in the diazepam solvent (the spectra of BSA and the BSA + DIA mixture without the addition of NPA are shown in Figure S2). Therefore, in all experiments (including experiments with WRF to standardize the methodology), from the obtained I_rel_ values of the reaction mixture we subtracted the value of I_rel_^0^, which is the relative integral intensity of the range 1.86–1.81 ppm of the spectrum of pure BSA (for control measurements) and the spectrum of the BSA-inhibitor mixture without substrate (for measurements with WRF or DIA). In other words, the relative integrated intensity of the range 1.86–1.81 ppm in the spectrum of BSA was taken as zero intensity in control measurements, the relative integrated intensity of the range 1.86–1.81 ppm in the spectrum of the BSA-inhibitor mixture was taken as zero intensity in measurements with the addition of an inhibitor (Figure S2). The experiment was repeated three times. Intensity values are presented as mean ± standard deviation. The measurement result is presented in Figure 9.

It has been revealed that WRF significantly inhibits the release of acetate during the hydrolysis of NPA by BSA (Figure 9A). Since WRF is a specific ligand of site Sudlow I, we have additionally confirmed our previous hypothesis [14] that site Sudlow I is the main site of true esterase activity of albumin. In the case of DIA, the plot of acetate yield in the presence of DIA lies below the control plot (Figure 9B), although the differences are not statistically significant. We do not exclude that DIA may have some allosteric effect on site Sudlow I, but it is not possible to detect this effect by NMR.

### 2.5. Effect of WRF and DIA on Albumin Esterase Activity Towards NPA According to Spectrophotometry

**Figure 10:**
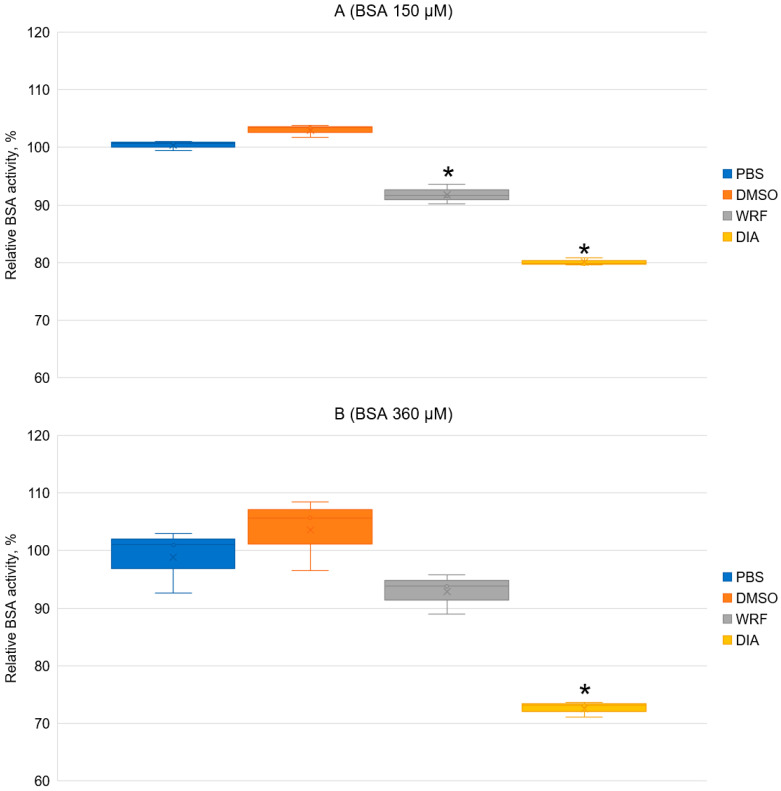
Effect of WRF and DIA on the esterase activity (stationary phase of nitrophenol yield) of BSA at concentrations of 150 μM (**A**) and 360 μM (**B**) towards NPA. Phosphate buffered-saline (PBS), control sample without inhibitors; DMSO, sample with the addition of a volume of DMSO equivalent to the volume of inhibitor solutions (10% of the total volume of the mixture); WRF, sample with the addition of WRF in a 2:1 ratio; DIA, sample with the addition of DIA in a ratio of 2:1; *, significance of difference from the control *p* < 0.05.

Previously, we studied the hydrolytic activity of albumin of different species in low concentrations (15 μM) towards NPA by detecting the yield of nitrophenol spectrophotometrically, and then performed inhibitory analysis of this activity [43]. We showed that WRF does not inhibit the release of nitrophenol at the pre-stationary phase of the reaction (pseudoesterase activity of site Sudlow II) and inhibits the release of nitrophenol at the stationary phase of the reaction (esterase activity of site Sudlow I). Ibuprofen (a ligand of site Sudlow II) inhibits the release of nitrophenol in the pre-stationary phase of the reaction, and inhibits the release of nitrophenol in a noncompetitive manner at the stationary phase of the reaction. Earlier, we applied the method of spectrophotometry to investigate the effect of CBDP and ethopropazine on true esterase activity of BSA [54]. Here, using spectrophotometry again, we studied the effect of WRF and DIA on true esterase activity (stationary phase of nitrophenol yield) of high concentrations of BSA (150 and 360 μM, which are comparable to the conditions of the bloodstream and to the conditions of the NMR experiment described above). The result of the experiment is shown in Figure 10.

In the experiment with 150 μM of BSA, WRF and DIA significantly inhibit the rate of nitrophenol yield (Figure 10A). The fact that specific ligand of site Sudlow I WRF reduces the yield of nitrophenol in the stationary phase additionally supports our hypothesis [14] that site Sudlow I is the site of true esterase activity of albumin. Specific ligand of site Sudlow II DIA reduces the rate of nitrophenol release in the stationary phase, which indicates that DIA may noncompetitively inhibit the true esterase activity at site Sudlow I.

It is interesting to note that at a BSA concentration of 360 μM (Figure 10B), only diazepam significantly inhibits nitrophenol yield. Conversely, the inhibitory activity of WRF weakens. We believe that this effect may be associated with the phenomenon of molecular crowding. As we mentioned in our earlier review [9], a distinctive feature of living systems is that biochemical processes occur in an environment containing high concentrations of macromolecules (50–400 mg/mL). Such conditions are called molecular crowding. Due to the dense medium, the volume of available solvent decreases, which leads to an increase in the effective concentration of macromolecules and an increase in their chemical activity. This in turn changes the rates and equilibrium constants of their reactions. In particular, this effect promotes the association of macromolecules, for example, the association of proteins into supramolecular structures. Crowding can also affect enzymatic reactions involving small molecules if the conformation of the enzyme changes during the reaction [55]. To date, the influence of molecular crowding on the structure of many proteins has been experimentally demonstrated [56,57,58], as well as on binding and the catalytic activity of a number of enzymes [59,60,61]. Lamy et al. showed that the kinetic constants for the hydrolysis of NPA by bovine albumin change with the increasing BSA concentration and during protein oligomerization [62]. It is interesting to note that, according to our experiment, the apparent molecular crowding has a greater effect on the interaction of BSA with WRF compared to DIA, that is, site Sudlow I is more susceptible to the influence of crowding compared to site Sudlow II.

Concluding, we showed spectrophotometrically that the specific ligand of site Sudlow I warfarin inhibits the true esterase activity of albumin towards NPA in this site. The specific ligand of site Sudlow II diazepam has an allosteric inhibitory effect on the true esterase activity of albumin in site Sudlow I. To reveal the molecular mechanism of this allosteric effect, we studied the interaction of NPA, WRF, and DIA with BSA using molecular modeling methods, which are discussed in the next section.

### 2.6. Effect of WRF and DIA on Albumin Esterase Activity Towards NPA According to Molecular Modeling

In the computational experiment, we studied the interaction of NPA with Sudlow sites of BSA in the absence and presence of WRF and DIA. At the first step, with the help of a molecular modeling procedure, we obtained four possible BSA complexes with the following substrate and inhibitors (Figure S3):

- Inhibitors are absent, NPA is bound in site Sudlow I (Figure S3A);
- DIA is bound in site Sudlow II, NPA is bound in site Sudlow I (Figure S3B);
- Inhibitors are absent, NPA is bound in site Sudlow II (Figure S3C);
- WRF is bound in site Sudlow I, NPA is bound in site Sudlow II (Figure S3D).

**Figure 11:**
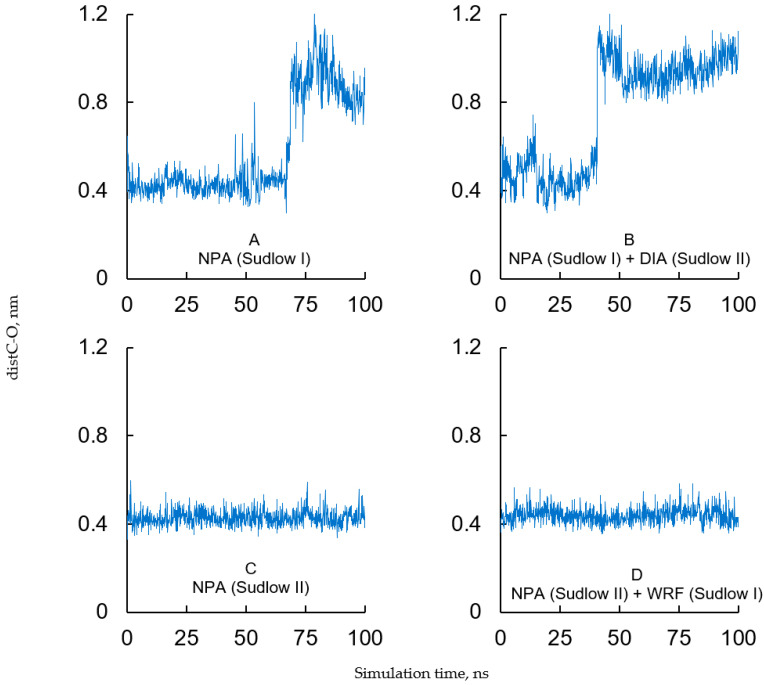
Time dependence of the distance (distC-O) between the carboxyl carbon of NPA and the hydroxyl oxygen of catalytic tyrosine (Tyr149 in site Sudlow I or Tyr410 in site Sudlow II of BSA). (**A**) Complex of BSA with NPA in site Sudlow I and empty site Sudlow II; (**B**) complex of BSA with NPA in site Sudlow I and DIA in site Sudlow II; (**C**) complex of BSA with NPA in site Sudlow II and empty site Sudlow I; (**D**) complex of BSA with NPA in site Sudlow II and WRF in site Sudlow I.

At the next step, the conformational behavior of the obtained complexes was modeled by 100 ns MD simulation. The obtained trajectories were used to plot the time dependences of the distance (distC-O) between the carboxyl carbon of NPA and the hydroxyl oxygen of catalytic tyrosine (Tyr149 or Tyr410) (Figure 11). The value of distC-O can help to evaluate if the conformation of NPA is productive (the conformation in which the attack of the hydroxyl group of the tyrosine on the carboxyl carbon is possible). The value of distC-O of about 0.4 nm (4 Å) is considered to be appropriate for nucleophilic attack [63].

In the complex of BSA with NPA in Sudlow I and free Sudlow II, the NPA molecule remains bound near Tyr149 throughout the 70 ns of the simulation; the value of distC-O fluctuates at approximately 0.4 nm (Figure 11A). For part of this period, the carboxyl oxygen of NPA interacts with the side chain of His241, the ether oxygen of NPA interacts with the side chain of Arg256, the nitro group of NPA interacts with the side chain of Arg198, and the aromatic ring of NPA interacts with the side chain of Tyr149 (Figure S4A). After 70 ns, the ligand molecule loses contact with Tyr149 and for the remaining 30 ns of the simulation it is bound at the entrance of the site Sudlow I (near amino acids Arg194, Arg198, Arg217, and Lys221, Figure S4B). Similar results were obtained in our earlier work when we modeled the interaction of NPA with site Sudlow I of free BSA using GROMOS53a6 force field [14].

In the complex of BSA with NPA in Sudlow I and DIA in Sudlow II, the position of the NPA molecule is less stable than in the absence of diazepam (Figure 11B). For 40 ns, NPA remains in site Sudlow I close to Tyr149; however, the position of the NPA molecule is different than in the case of diazepam-free BSA. Thus, the carboxyl oxygen of NPA interacts with Arg256 instead of His241, the ether oxygen of NPA interacts with His241 instead of Arg256, and the π-π bond between the aromatic rings of NPA and Tyr149 is lost after 1 ns of the simulation. After 40 ns of the simulation, the NPA molecule moves away from Tyr149 and for the remaining 60 ns of the simulation it is bound at the entrance of Sudlow I (near amino acids Arg194, Arg198, Arg217 and Lys221). Thus, according to molecular modeling, DIA destabilizes the productive conformation of NPA in site Sudlow I, thereby inhibiting the true esterase activity of albumin through a noncompetitive mechanism.

This is consistent with our experimental data. As noted above in Section 2.4, according to NMR spectroscopy, diazepam had some inhibitory effect on the yield of acetate, but this was statistically insignificant (possibly due to limitations of the method). According to our previously published data obtained spectrophotometrically in vitro, ibuprofen (another ligand of site Sudlow II) also inhibited the true esterase activity of albumins of different species in a noncompetitive manner [43]. Finally, Section 2.5 describes a spectrophotometric experiment with high concentrations of BSA, according to which DIA noncompetitively inhibits the true esterase activity of albumin in site Sudlow I.

In the complex of BSA with NPA in site Sudlow II and the empty site Sudlow I, the NPA molecule remains in the vicinity of catalytic Tyr410 throughout the simulation; the value of distC-O is fluctuating consistently at approximately 0.4 nm (Figure 11C). The carboxyl oxygen of NPA remains in the vicinity of Ser488 either forming a hydrogen bond with its hydroxyl group (Figure S4C) or interacting through a water molecule (Figure S4D). The aromatic rings of NPA and Tyr410 form π-π interaction (Figure S4C,D). Findings from this computational experiment are consistent with multiple in vitro experiments indicating that Tyr410 (Tyr411 in HSA) is the most reactive residue when albumin is interacting with esters. Similar results were obtained in our earlier work when we modeled the interaction of NPA with site Sudlow II of free BSA using GROMOS53a6 force field [14]. Binding of WRF in site Sudlow I does not affect the binding of the ligand in site Sudlow II (Figure 11D); the NPA molecule remains bound near Tyr410 throughout the simulation in the same conformation as in the case of warfarin-free BSA, which means that WRF bound in site Sudlow I has no allosteric effect on the interaction of NPA with site Sudlow II. This result provides an additional confirmation that the slowdown in the acetate yield during the interaction of BSA with NPA in the presence of WRF in the NMR experiment, as well as the slowdown in the nitrophenol yield in the spectrophotometric experiment, is associated with the competitive inhibition of the reaction in site Sudlow I by warfarin.

**Figure 12:**
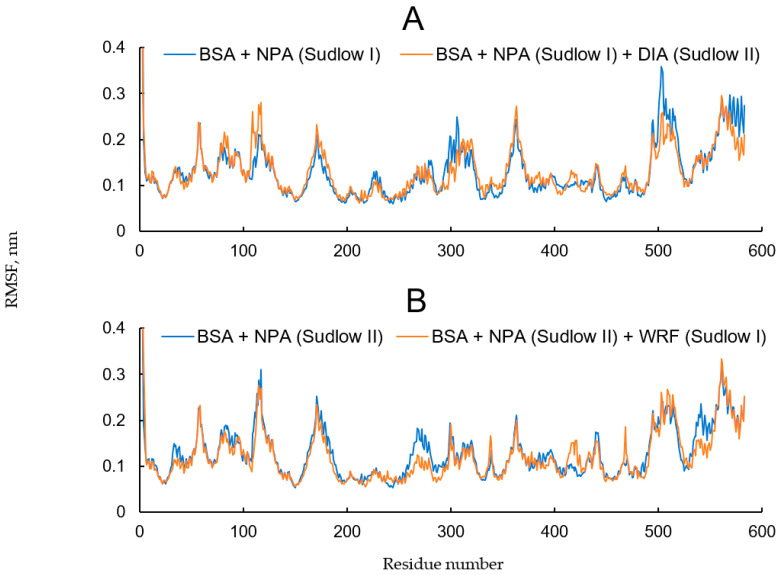
Root mean square fluctuation (RMSF) of the Cα-atoms of BSA complexes with ligands and inhibitors. (**A**) NPA is bound in site Sudlow I and site Sudlow II is empty or interacts with DIA; (**B**) NPA is bound in site Sudlow II and Sudlow I site is empty or interacts with WRF.

We calculated the root mean square fluctuation (RMSF) of the C^α^-atoms of BSA during the simulations (Figure 12). The RMSF value shows which moieties of BSA are most mobile during the simulation.

**Figure 13:**
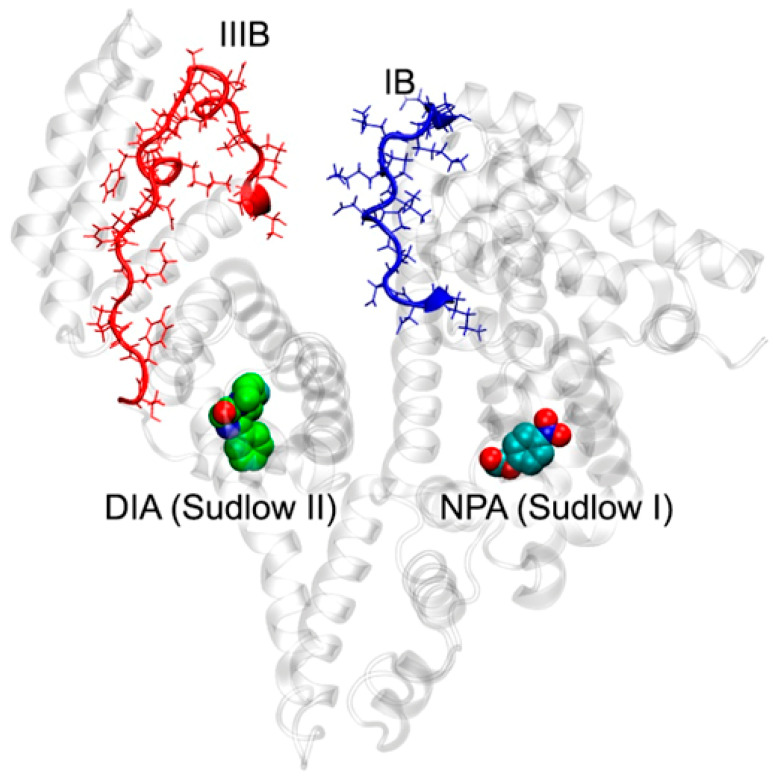
Interaction between subdomains IB and IIIB of BSA in its complex with NPA and DIA according to MD simulation. The regions most affected by DIA binding are highlighted blue and red (Lys106–Pro119 in domain IB and Thr495–Glu519 in domain IIIB). Cyan, blue and red spheres represent carbon, nitrogen and oxygen atoms, respectively. Carbon atoms of DIA are shown in green.

Comparing the diagrams in Figure 12A allows us to determine how diazepam binding in site Sudlow II affects the conformational mobility of BSA. In the presence of diazepam, the conformational mobility of domain IB increases, while the mobility of domain IIIB decreases. The greatest changes are observed for BSA moieties consisting of amino acids 106–119 and 495–519 (Figure 12A). These fragments are highlighted in Figure 13, which represents the conformation of the BSA–NPA–DIA complex at the final point of the simulation. As can be seen from Figure 13, these fragments belong to different ends of the albumin polypeptide chain; however, in a three-dimensional space, they are located close to each other. The interaction between these regions maintains the three-dimensional structure of the protein in aqueous solution. Diazepam binding in site Sudlow II limits the conformational mobility of the region Thr495–Glu519 (highlighted red in Figure 13), due to which the region Lys106–Pro119 (highlighted blue in Figure 13) is forced to change its conformation to keep the connection with the region Thr495–Glu519, and this affects the position of NPA in site Sudlow I. A more detailed analysis showed that binding of DIA in site Sudlow II “pushes apart” the residues of the site, including Phe487 and, along with it, the entire fragment Phe487–Asp493. Because of this shift, the side chain of Asp493 forms a hydrogen bond with the NH group of the backbone of Thr495 at 45 ns of the simulation. In the case of absence of DIA in site Sudlow II, such a bond is also formed during the simulation; however, later, at 65 ns of the simulation. It is interesting to note that the moment the NPA moves away from Tyr149 (Figure 11A,B) approximately coincides with the moment of the formation of the hydrogen bond between Asp493 and Thr495. We believe that the Asp493–Thr495 interaction may restrict the movement of the Thr495–Glu519 region (highlighted red in Figure 13), which in turn enhances the conformational mobility of the Lys106–Pro119 region (highlighted blue in Figure 13), and, as a consequence, changes the conformation of site Sudlow I and distances NPA from Tyr149.

Binding of WRF in site Sudlow I has much less effect on the conformational behavior of the BSA globule and on interaction of NPA with site Sudlow II (Figure 12B).

## 3. Discussion

Based on the results of our in silico experiments devoted to the interaction of HSA, BSA, and RSA with WRF and DIA, it can be concluded that the binding activity of albumins towards WRF differs between HSA and RSA to a lesser extent compared to BSA. Binding poses of WRF in site Sudlow I of HSA and RSA are more similar to each other compared to the binding pose of WRF in site Sudlow I of BSA (Table 1, Table 2 and Table 3). This finding is consistent with known experimental data. Thus, Poór et al. measured the stability of complexes of HSA, BSA, and RSA with some ligands of site Sudlow I (including WRF) in vitro and, according to their results, the features of albumin–ligand complexes were also more similar for HSA and RSA compared to BSA [33]. Brown and Muller used the circular dichroism method to show that WRF interacts with BSA more efficiently than with HSA [64]. A similar result was obtained by Ràfols et al. using isothermal titration calorimetry [65].

According to our results, in terms of binding activity, site Sudlow II is more conservative compared to site Sudlow I for all studied species; the binding modes of DIA are similar for HSA, BSA, and RSA. As for the comparative analysis with known experimental data, we highlighted the work of Pistolozzi and Bertucci [66], who studied the binding of various ligands (including DIA) to human, bovine, rat, and dog albumins using the circular dichroism method. According to the data obtained in this work, diazepam bound slightly more strongly to HSA compared to BSA and RSA; however, the difference was not as pronounced as in the case of warfarin in the experiments of Brown and Muller [64].

Without doubt, these interspecies differences in albumin binding activity are associated with structural differences in the structure of the ligand–binding sites of HSA, BSA, and RSA, which result from processes of species adaptation to variable environmental conditions. The greater variability of site Sudlow I compared to Sudlow II can be explained by the fact that the former is larger and contains more polar residues, mutations which can have a greater effect on the affinity of the ligands compared to nonpolar amino acids. In our earlier review, we compared the structural differences in HSA, BSA, and RSA binding sites in more detail [1].

According to our experiments in vitro presented here, WRF (a ligand of site Sudlow I) inhibits the yield of nitrophenol in the stationary phase of the reaction (esterase activity of site Sudlow I), which supports our hypothesis that site Sudlow I is the site of the true esterase activity of albumin. In one of our early works, we showed that WRF had no effect on pseudoesterase activity of site Sudlow II [43]. According to the computational experiments presented here, WRF has no allosteric effect on the functioning of site Sudlow II. Here, we used a racemic mixture of WRF for in vitro experiments, since anticoagulant medication warfarin, which we used in our earlier in vivo studies [30] (the details are described below), is a racemic mixture, and we wanted to bring our experiments closer to in vivo conditions whenever possible. At 25 °C and 37 °C, the association constant for the binding of S-WRF to HSA is 1.3 and 1.2 times higher compared to R-WRF [67], respectively. However, two enantiomers of WRF adopt very similar conformations when bound to HSA and make many of the same specific contacts with amino acid side chains in site Sudlow I [68]. In our experiments, we used saturating concentrations of inhibitors, so we believe that the use of a non-racemic mixture would not affect the qualitative picture of the interaction of WRF with albumins nor its ability to inhibit the esterase activity of the protein. Although, the values of the inhibition constants would definitely differ for the R- and S-enantiomers.

In our study [43], we found that ibuprofen can inhibit the true esterase reaction in site Sudlow I in a noncompetitive manner. Here, in spectrophotometric and computational experiments, we have obtained a similar result for diazepam. The totality of the data obtained allows us to conclude that site Sudlow I is more susceptible to allosteric modulation compared to site Sudlow II, and one of the key roles in this process is played by the mobility of domain IB during its interaction with domain IIIB.

The role of domains I and III in the physiological role of albumin has been described by other researchers. Thus, Paar et al. [69] studied the structural features of albumin in patients with chronic liver disease. The albumin of these patients was found to be oxidized and overloaded with fatty acids and bilirubin. The authors demonstrated that conformation and flexibility of patient HSA were changed compared to healthy donor HSA. The latter has an inherent flexibility with the dynamic movement of domains I and III. The MD study showed that the motion of domains I and III, caused by the binding of myristate molecules, increased the radius of gyration of HSA [70]. Ketrat et al. employed MD simulations to reveal structural and dynamic properties of canine serum albumin compared to BSA and HSA and showed that the dynamics of domains I and III define the characteristics of each albumin [71].

Analysis of the molecular mechanisms of the influence of various ligands on the esterase activity of albumin and the role of species differences in these effects is not only of fundamental, but also of applied importance. For example, targeting albumin with molecules that modulate its binding and/or esterase activity may become a way to enhance the detoxification of OPs in the bloodstream. Two fundamentally different ways of modulating albumin are possible: (1) increasing the affinity of OPs for sites of protein (pseudo)esterase activity thus enhancing their detoxification by albumin; (2) weakening of the affinity of OPs to sites of albumin (pseudo)esterase activity with their subsequent detoxification by blood esterases (cholinesterases, CES) through stoichiometric interaction. The first way could be productive for both highly toxic and low-toxic OPs. The second way is applicable only to highly toxic OPs in the case of poisoning with sublethal doses (less than LD_50_). Earlier, we performed preclinical testing of the effect of modulators of albumin binding and esterase activity on the survival of rats during acute poisoning with pinacolyl methylphosphonofluoridate (PMFP) [30]. According to the data obtained, WRF did not affect the survival of rodents, while ibuprofen increased the death of animals. Epigallocatechin gallate (EGCG, the major polyphenol of the decaffeinated green tea extract), which can bind to both Sudlow sites [72], promoted the survival of rats in the first hours after poisoning. Evaluation of the degree of inhibition of BChE and CES in the blood plasma of rats, one day after poisoning, revealed a statistically significant decrease in the activity of BChE and CES in rats that were administered EGCG, compared to positive control rats. The data obtained indicate that EGCG, reversibly interacting with albumin, increases the likelihood of the stoichiometric interaction of PMFP with blood plasma esterases, enhancing the detoxification of OPs. Here, we showed that the features of ligand binding to HSA and RSA are close to each other. Consequently, preclinical testing of modulators of albumin activity, as well as other in vivo experiments connected with albumin, can be extrapolated to human organisms.

In conclusion, we would like to sum up our long-term studies on the (pseudo)esterase activity of albumin. With the help of the NMR technique, we demonstrated albumin possesses true esterase activity towards NPA and other esters. We hypothesized that that the site of true esterase activity does not coincide with the known site of pseudoesterase activity Sudlow II. Using computational modeling, we substantiated that site Sudlow I with the catalytic tyrosine Tyr150 (Tyr149 in BSA) is responsible for true esterase activity [14]. In addition, we proposed a scheme for the hydrolysis of NPA by albumin (Scheme 1) that describes the entire hydrolytic activity of albumin as a whole in both sites Sudlow, as well as the functioning of the sites of pseudoesterase and true esterase activity of the protein separately [43]. The values of the kinetic constants of (pseudo)esterase activity of human, bovine, and rat albumins towards NPA and POX were determined quantitatively using spectrophotometry [43].

In a range of computational experiments with the help of the molecular docking procedure and the method of MD, we studied the binding of NPA and POX to Sudlow sites of albumins of different species and showed that the productive conformation of NPA and POX in site Sudlow II is much more stable than in site Sudlow I [14,73]. The stability of this productive conformation (the conformation in which a nucleophilic attack of catalytic tyrosine on the substrate is possible) determines the efficiency of the first stages of the esterase reaction, i.e., stage of the ES complex formation and stage of the acetylation/phosphorylation of a catalytic tyrosine (Scheme 1). That is, the acetylation/phosphorylation step of site Sudlow II is faster compared to Sudlow I. Undoubtedly, this effect is due to the fact that site Sudlow II is smaller and includes predominantly nonpolar residues with a single polar region containing Tyr411 and Arg410. This structure makes site Sudlow II an ideal binding pocket for NPA and POX.

In our study [74], using the MD method and the quantum-chemical semi-empirical AM1 method, we studied the mechanism of organophosphate release from HSA and BChE adducts when exposed to fluoride ion. According to the assessment of the efficiency of the P-O bond cleavage proposed by us, the adduct of POX with Tyr150 is cleaved more efficiently than the adduct with Tyr411. The obtained result suggests that the last stage of albumin esterase activity (i.e., the stage of deacetylation or dephosphorylation), proceeds faster in site Sudlow I, which again indicates that this site is the site of the true esterase activity of HSA. The conformation of organophosphate adducts formed at Sudlow site I is more similar to the active site of inhibited BChE than that of adducts formed at Sudlow site II. Thus, in the adduct of POX with Tyr150 of HSA, the imidazole group of His242 interacts with the phosphoryl oxygen of the organophosphate moiety (i.e., His242 serves as the oxyanion hole), and the side chain of Arg257 forms a hydrogen bond with one of the ether oxygen atoms of the organophosphate moiety (i.e., Arg257 plays the same role that His438 plays is in the active site of inhibited BChE) [74]. Due to this feature, the reaction of deacetylation/dephosphorylation occurs faster in site Sudlow I compared to Sudlow II.

And finally, the inhibitory analysis of the hydrolysis of NPA and POX in sites Sudlow I and II, which we performed earlier using spectrophotometry [43] and which we presented here using NMR spectroscopy and MD simulation, as well as some of our studies on allosteric modulation of albumin esterase activity [75,76,77,78], complete this cycle of research. However, the effect of endogenous ligands of site III in subdomain IB (e.g., hemin [79]) on the esterase activity of albumin is unexplored, and this is the focus of our future research.

The methodological approach that we proposed, which combines NMR spectroscopy for the detection of non-chromogenic reaction products, the method of spectrophotometry for quantitative evaluation of kinetic constants, and in silico tools for establishing the molecular mechanisms of substrate–enzyme interaction, allowed us to outline a sketch of the mechanism of the pseudo- and true esterase activity of albumin. The developed method will make it possible to more specifically study the mechanisms of other enzymatic activities of albumin in the future, both already known [7] and those that have yet to be discovered.

## 4. Methods

### 4.1. Chemicals

Diazepam (5 mg/mL) in solution form for injections and warfarin sodium salt (with the main component content no less than 90% according to NMR analysis) was provided by the Research Institute of Hygiene, Occupational Pathology and Human Ecology. Warfarin stock solution was prepared by dissolving the salt in DMSO (for spectrofluorimetric experiments) or in deuterated dimethyl sulfoxide (DMSO d6, for NMR experiments); dissolution was carried out in a thermostat at a temperature of 37 °C. DMSO d6 and deuterated water were provided by the Centre for Magnetic Resonance of St. Petersburg State University. Phosphate buffered-saline (PBS, pH 7.4) was purchased from Biolot (St. Petersburg, Russia). All other chemicals were purchased from Sigma-Aldrich (St. Louis, MO, USA). Globulin free and fatty acid free BSA purchased from Sigma-Aldrich (St. Louis, MO, USA) was used.

### 4.2. Spectrofluorometry

On the day of the experiment, a solution of BSA in PBS was prepared, which was then mixed with solutions of WRF and DIA and centrifuged for 90 s at 14,000 rpm. The final concentration of BSA in all samples was 1 mg/mL (15 µM), the concentrations of WRF and DIA were 120.0, 92.3, 71.0, 54.6, 42.0, 32.3, 24.9, 14.7, 0 µM. Solutions of WRF and DIA of the same concentrations were prepared in PBS as the references. Before recording the spectra, the solutions were incubated for 30 min in a thermostat at temperatures of 25° or 37 °C. Spectra were recorded in 0.4 cm quartz cuvette (Hellma Analytics, Müllheim, Germany) at room temperature (22 °C) at the Physics faculty of St. Petersburg University using spectrofluorophotometer RF-6000 (Shimadzu, Kyoto, Japan). Luminescence excitation for all samples was performed at 280 nm. The bandpass for excitation and emission was set at 5 nm and emission spectra were corrected for instrument sensitivity. The experiment for each ligand was performed in triplicate with one reference for all replicates.

### 4.3. NMR Spectroscopy

The methodology of NMR experiments was the same as in our previous studies [14,54]. On the day of the experiment, a solution of BSA in a mixture of PBS and deuterated water (9:1), a solution of the internal standard DSS in bi-distilled water and solutions of NPA, and warfarin and diazepam in DMSO d6 were prepared. Solutions of inhibitors and BSA were mixed and incubated for 30 min at room temperature (25 °C). An equivalent volume of DMSO d6 was added to the control sample without inhibitors (thus, the volume of DMSO d6 was identical in each sample, 5% of the total volume of the mixture). Then, immediately before recording the spectrum, the substrate solution was added to the reaction mixture. In the final reaction mixture, the BSA concentration was 180 μM, the NPA concentration was 3.6 mM, the warfarin and diazepam concentrations were 360 μM. The prepared samples were scanned by ^1^H NMR in a Bruker Avance III 500 NMR spectrometer at room temperature (25 °C). To suppress the water signal, the excitation sculpting method with “perfect echo” for cleaning phase distortions was used [80]. The spectra were recorded every 3 min. At each time point, 32 scans were performed. The relaxation delay was set to 1 s. Chemical shifts δ of solution components accurate to 0.001 ppm were calibrated against tetramethylsilane Si(CH_3_)_4_. DSS and DMSO were used as the internal standards for the experiments with warfarin and diazepam, respectively. The relative integral intensity (I_rel_) of the signals of NPA and the products were calculated as the ratio of their integrated signal intensity to the integrated signal intensity of the internal standard. The reference relative integral intensity I_rel_^0^ was calculated as I_rel_ for the region 1.86–1.81 ppm (corresponding to the signal of acetate) of the spectrum of pure BSA free of the inhibitors and NPA (for control measurements) or the spectrum of a mixture of BSA-inhibitor free of NPA (for measurements with WRF or DIA). Each experiment was repeated three times, then the dependence of I_rel_-I_rel_^0^ as the mean ± SD on time was plotted.

### 4.4. Spectrophotometry

The methodology of spectrophotometric experiments was the same as in our earlier work [54]. The experiment was performed in the mode of preliminary incubation of a test inhibitor in a BSA solution in PBS for 30 min at a temperature of 25 °C. The concentrations of BSA, WRF, DIA, and NPA in the reaction mixture were 150, 300, 300, and 3000 µM in experiment No.1 and 360, 720, 720, and 7200 µM in experiment No.2, respectively. As a control, we used a reaction mixture of BSA and NPA without the addition of inhibitors and with the addition of a volume of DMSO equivalent to the volume of inhibitor solutions (10% of the total volume of the mixture). The concentration of nitrophenol was determined spectrophotometrically (405 nm) over 15 min. The experiment was carried out in triplicate. The obtained data were subjected to primary processing in MS Excel. For the calculation, we used the change in absorption from 267 to 360 s of the experiment (stationary phase of the reaction). The arithmetic mean between blank samples with PBS and DMSO was taken as 100% activity. The statistical significance of differences was assessed using Student’s *t*-test.

### 4.5. Molecular Modeling

The methodology of molecular modeling was similar to our previous computational experiments with slight modifications [14,54].

#### 4.5.1. Three-Dimensional Models Preparation

The crystal structures of HSA and BSA from the Protein Data Bank [81] were used as the three-dimensional models of human and bovine albumin. Structure 2BXD [5] was used for WRF docking into site Sudlow I of HSA, structure 2BXF [5] for docking of DIA into site Sudlow II of HSA, and structure 4JK4 [26] for the docking of WRF and DIA into sites Sudlow I and II of BSA. Water and ligand molecules were removed from the structures and missing atoms were completed using Visual Molecular Dynamics v.1.9.4a53 (VMD, University of Illinois Urbana-Champaign, Champaign, IL, USA) [82]. A three-dimensional model of RSA was built by us earlier using the method of homology modeling [47]. A three-dimensional model of NPA was built and optimized by the method of energy minimization in vacuum using Avogadro v1.2.0 software package [83]. Three-dimensional models of WRF and DIA were retrieved from 2BXD and 2BXF structures.

#### 4.5.2. Molecular Docking

The docking of NPA, WRF, and DIA into the binding sites of HSA, BSA, and RSA was performed using Autodock Vina 1.1.2 software package [84]. In the studied protein binding site, a search area of 20 × 20 × 20 Å^3^ was set. The number of runs (exhaustiveness) was ten. The allowable maximum scatter of conformational energies in the output file (energy_range) was taken to be 3 kcal/mol. The number of the most optimal (energetically favorable) conformations in the output file (num_modes) was set to ten. The protein molecule was set to be rigid, and the conformation of the ligands could vary. The result of each docking procedure was a set of the ten most energetically favorable conformations. In the case of the docking of NPA, we selected the conformation of the protein-–ligand complex with the minimum distance between the carbonyl atom of the ligand and the oxygen atom of the hydroxyl group of the catalytic amino acid. In the case of the docking of WRF and DIA, the most energetically favorable conformations were chosen.

#### 4.5.3. Molecular Dynamics

Conformational changes in ligand–albumin complexes were calculated by MD simulation using GROMACS2022.2 software (University of Groningen, Groningen, The Netherlands) [85] and CHARMM27 force field [86]. Parameters for the ligands were obtained with the help of CHARMM-GUI Ligand Reader and Modeler (https://www.charmm-gui.org/, accessed on 8 October 2024) [87]. The complexes were placed virtually into a cubic periodic box filled with water molecules. The TIP3P water model (transferable intermolecular potential with three points) was used to describe water molecules [88]. To neutralize a system, sodium ions were added. Temperature (300 K) and pressure (1 bar) were kept constant using a V-rescale thermostat [89] and a Parrinello–Rahman barostat [90], with coupling constants of 0.1 ps and 2.0 ps, respectively. Long-range electrostatic interactions were treated by the particle-mesh Ewald method [91]. Lennard-Jones interactions were calculated with a cutoff of 1.0 nm. The LINCS algorithm (linear constraint solver for molecular simulations) was used to constrain bonds length [92]. Before running the MD simulations, all the structures were minimized by steepest descent energy minimization and equilibrated under NVT (1000 ps) and NPT (5000 ps) ensembles. The time step for MD simulation was 0.002 ps. The simulation length was 50 ns for the complexes of HSA, BSA and RSA with WRF and DIA, and 100 ns for the complexes of BSA with NPA (both in the absence and presence of WRF and DIA). MD simulation was performed in the supercomputer center of Peter the Great St. Petersburg Polytechnic University (https://scc.spbstu.ru, accessed on 8 October 2024).

## 5. Conclusions

In the presented study, we investigated the features of the interaction of specific ligands of Sudlow sites I and II of warfarin and diazepam with human, bovine, and rat albumins in vitro and in silico. It has been shown that the binding characteristics of human and rat serum albumin differ to a lesser extent compared to bovine albumin. Interspecies differences are more pronounced for site Sudlow I compared to site Sudlow II. The results of molecular modeling were compared with known experimental data on the interaction of albumin with WRF and DIA. Their consistency suggests that the methods of molecular modeling can be used to study the molecular mechanisms of the functional properties of albumin.

In the next step, using NMR spectroscopy and spectrophotometry, we determined how WRF and DIA affect the yield of acetate and nitrophenol when NPA interacts with high concentrations of BSA, and compared the data obtained with our earlier studies on the effect of WRF and ibuprofen (another ligand of site Sudlow II) on interaction of NPA with low concentrations of HSA, BSA, and RSA. Using the method of MD, we studied the molecular mechanisms of the allosteric effect of WRF and DIA on the interaction of BSA with NPA. The totality of all the data obtained indicates that WRF competitively inhibits the true esterase activity of site Sudlow I towards NPA, and DIA competitively inhibits the pseudoesterase activity of site Sudlow site II. WRF does not have an allosteric effect on site Sudlow II, whereas DIA and ibuprofen can inhibit the true esterase reaction in site Sudlow I in a noncompetitive manner. Thus, site Sudlow I is more susceptible to allosteric modulation compared to site Sudlow II, and one of the key roles in this process is played by the mobility of domain IB during its interaction with domain IIIB.

## Figures and Tables

**Table 1:** Root mean square deviation (RMSD, Å) between the X-ray and docked ligand poses and distances (Å) between functionally significant atoms of WRF, DIA, and the residues of sites Sudlow I and II of HSA according to X-ray, molecular docking and molecular dynamics (MD) data.

	X-Ray	Docking	Average Distance for MD Trajectory	Final Distance After 50 ns of MD Simulation	Lifetime of Direct Interaction, % of the Simulation
WRF in site Sudlow I					
RMSD X-ray/docked	0.85				
O^1^(WRF)-N^η1^(Arg222)	3.1	3.0	4.0	4.4	56%
O^2^(WRF)-O^η^(Tyr150)	3.0	3.5	6.6	6.4	1%
O^3^(WRF)-N^η2^(Arg222)	3.4	6.1	5.5	3.9	20%
O^3^(WRF)-N^ζ^(Lys199)	4.1	3.2	4.1	5.9	70%
DIA in site Sudlow II					
RMSD X-ray/docked	0.79				
O^1^(DIA)-O^η^(Tyr411)	3.1	4.7	5.0	6.2	22%
CL^2^(DIA)-S^γ^(Cys392)	3.5	4.1	4.3	4.1	70%
C^3^(DIA)-C^δ^(Arg485)	4.3	4.3	4.5	4.5	98%

Atoms O^1^(WRF), O^2^(WRF), O^3^(WRF), N^η1^(Arg222), O^η^(Tyr150), N^η2^(Arg222), O^1^(DIA), CL^2^(DIA), C^3^(DIA), O^η^(Tyr411), S^γ^(Cys392) and C^δ^(Arg485) are designated in Figure 2 and Figure 3.

**Table 2:** Distances (Å) between functionally significant atoms of WRF, DIA, and BSA, according to molecular docking and MD simulation.

	Docking	Average Value for MD Trajectory	Final Value After 50 ns of MD Simulation	Lifetime of Direct Interaction, % of the Simulation
WRF in site Sudlow I				
O^1^(WRF)-N^η1^(Arg217)	2.9	9.4	11.1	0.2%
O^2^(WRF)-N^η2^(Arg256)	2.4	7.3	7.9	0.2%
O^4^(WRF)-N^η2^(Arg198)	3.1	6.8	6.3	3%
O^3^(WRF)-N^ε^(Arg256)	4.4	4.2	3.5	69%
DIA in site Sudlow II				
O^1^(DIA)-O^η^(Tyr410)	2.7	5.4	3.3	25%
CL^2^(DIA)-S^γ^(Cys391)	4.2	4.5	4.2	54%
C^3^(DIA)-C^δ^(Arg484)	4.1	5.0	4.5	100%

Atoms O^1^(WRF), O^2^(WRF), O^3^(WRF), O^4^(WRF), N^η1^(Arg217), N^η2^(Arg256), N^η2^(Arg198), N^ε^(Arg256), O^1^(DIA), CL^2^(DIA), C^3^(DIA), O^η^(Tyr410), S^γ^(Cys391) and C^δ^(Arg484) are designated in Figure 5 and Figure 6.

**Table 3:** Distances (Å) between functionally significant atoms of WRF, DIA, and amino acids of the sites Sudlow I and II of RSA according to molecular docking and MD simulation.

	Docking	Average Value for MD Trajectory	Final Value After 50 ns of MD Simulation	Lifetime of Direct Interaction, % of the Simulation
WRF in site Sudlow I				
O^1^(WRF)-N^η1^(Arg222)	2.7	9.7	11.5	0.2%
O^2^(WRF)-O^η^(Tyr150)	2.7	4.7	3.1	50%
O^3^(WRF)-N^δ2^(Asn242)	6.8	6.0	3.4	9%
O^3^(WRF)-N^ζ^(Lys199)	3.2	5.2	4.8	16%
DIA in site Sudlow II				
O^1^(DIA)-O^η^(Tyr411)	3.7	5.9	2.9	30%
CL^2^(DIA)-S^γ^(Cys391)	3.9	4.7	3.7	52%
C^3^(DIA)-C^δ^(Arg485)	3.9	5.6	6.0	78%

Atoms O^1^(WRF), O^2^(WRF), O^3^(WRF), N^η1^(Arg222), O^η^(Tyr150), N^δ2^(Asn242), N^ζ^(Lys199), O^1^(DIA), CL^2^(DIA), C^3^(DIA), O^η^(Tyr411), S^γ^(Cys392), and C^δ^(Arg485) are designated in Figure 7 and Figure 8.

## Data Availability

The data presented in this study are available from the corresponding authors upon reasonable request.

## Supplementary Materials

The following supporting information can be downloaded at: https://www.mdpi.com/article/10.3390/ijms252111543/s1.

## Abbreviations

^1^H NMR	proton nuclear magnetic resonance
BChE	butyrylcholinesterase
BSA	bovine serum albumin
CES	carboxylesterase
DIA	diazepam
distC-O	the distance between the carboxyl carbon of NPA and the hydroxyl oxygen of catalytic tyrosine of albumin
DMSO	dimethyl sulfoxide
DMSO d6	deuterated dimethyl sulfoxide
DOPE	discrete optimized protein energy
DSS	sodium trimethylsilylpropanesulfonate
HSA	human serum albumin
I_rel_	relative integral intensity
I_rel_^0^	relative integral intensity of the range 1.86–1.81 ppm of the spectrum of pure BSA (for control measurements) and the spectrum of the BSA-inhibitor mixture without substrate (for measurements with WRF or DIA)
MD	molecular dynamics
NPA	p-nitrophenyl acetate
OPs	organophosphates
PBS	phosphate buffered-saline
PDB	protein data bank
PMFP	pinacolyl methylphosphonofluoridate
POX	paraoxon
RSA	rat serum albumin
WRF	warfarin
