# Supplementary material for: Modulation of Albumin Esterase Activity by Warfarin and Diazepam
Source: Int J Mol Sci. 2024 Oct 27;25(21):11543. doi: 10.3390/ijms252111543 (PMC11546595; doi:10.3390/ijms252111543)
Supplement: Supplementary file 1 [file ijms-25-11543-s001.zip › ijms-3237733-supplementary.pdf]

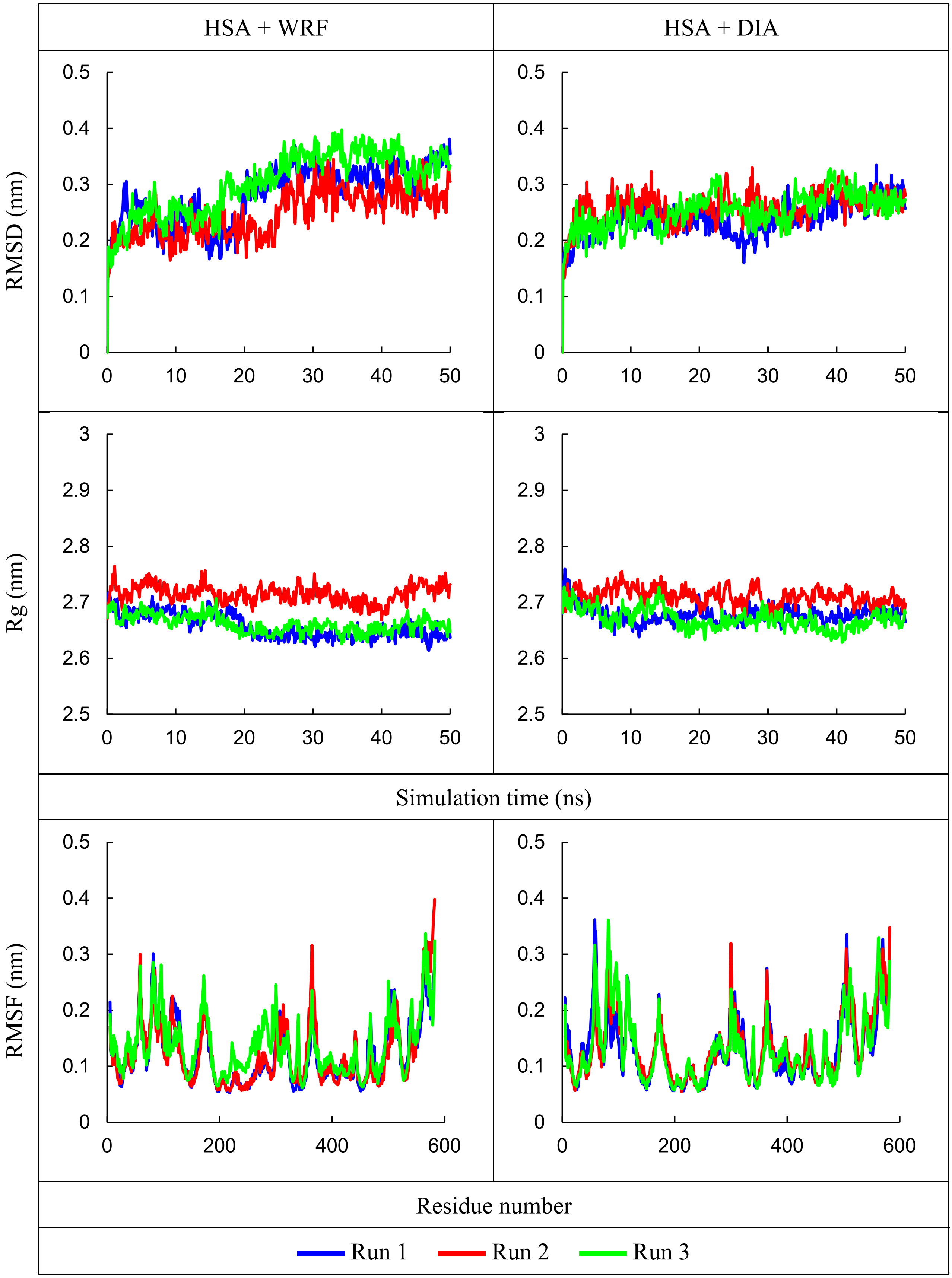

Figure S1. Conformation characteristics of the complexes of human serum albumin (HSA) with R-warfarin (WRF, left column) and diazepam (DIA, right column) calculated for the C^{α}-atoms of HSA. RMSD, root mean square deviation; Rg, radius of gyration; RMSF, root mean square fluctuation. For each ligand, the simulation was run three times, data for runs 1, 2 and 3 are shown with blue, red and green, respectively.

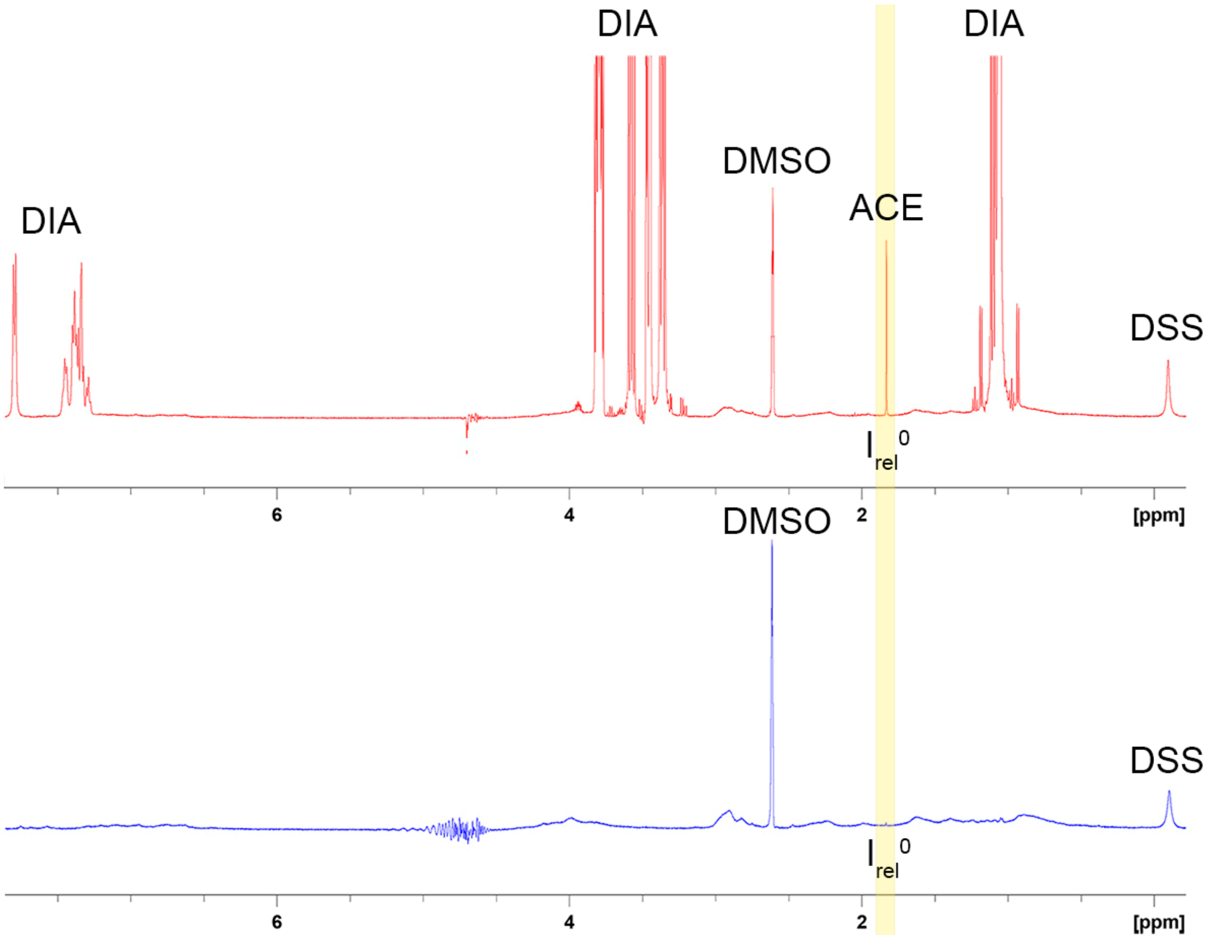

Figure S2. NMR spectra of solution of BSA (blue) and mixture of BSA with diazepam (DIA, red). Signal peaks for sodium trimethylsilylpropanesulfonate (DSS), dimethyl sulfoxide (DMSO), acetate group (ACE), and DIA solution are indicated. The chemical shift region corresponding to the signal of ACE is highlighted yellow. In NMR experiments aimed at analyzing the effect of inhibitors on the esterase activity of BSA, the relative integral intensity of this region was taken as zero intensity.

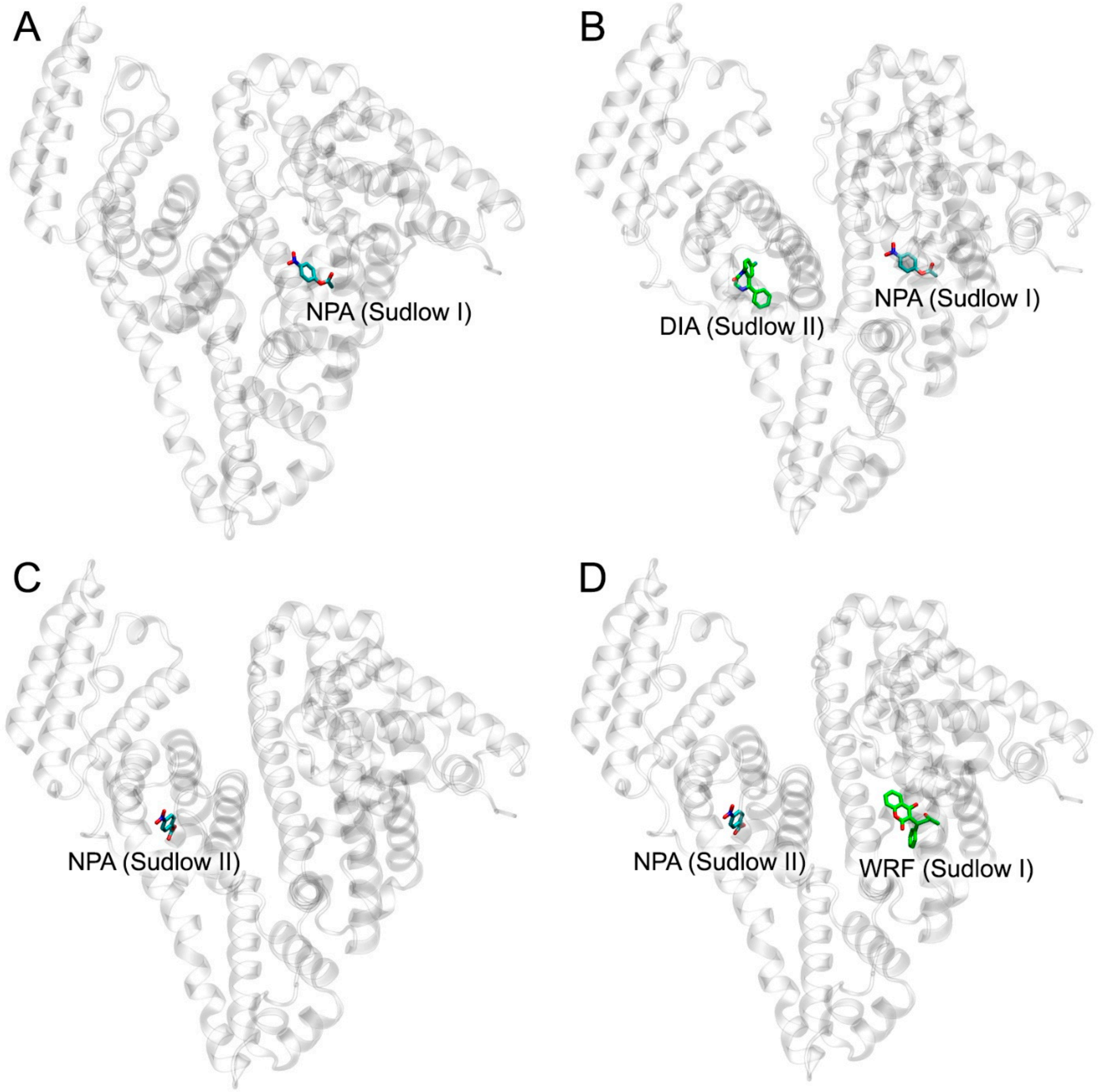

Figure S3. BSA complexes with nitrophenyl acetate (NPA) in sites Sudlow I (A, B) and Sudlow II (C, D), DIA in site Sudlow II (B), and WRF in site Sudlow I (D).

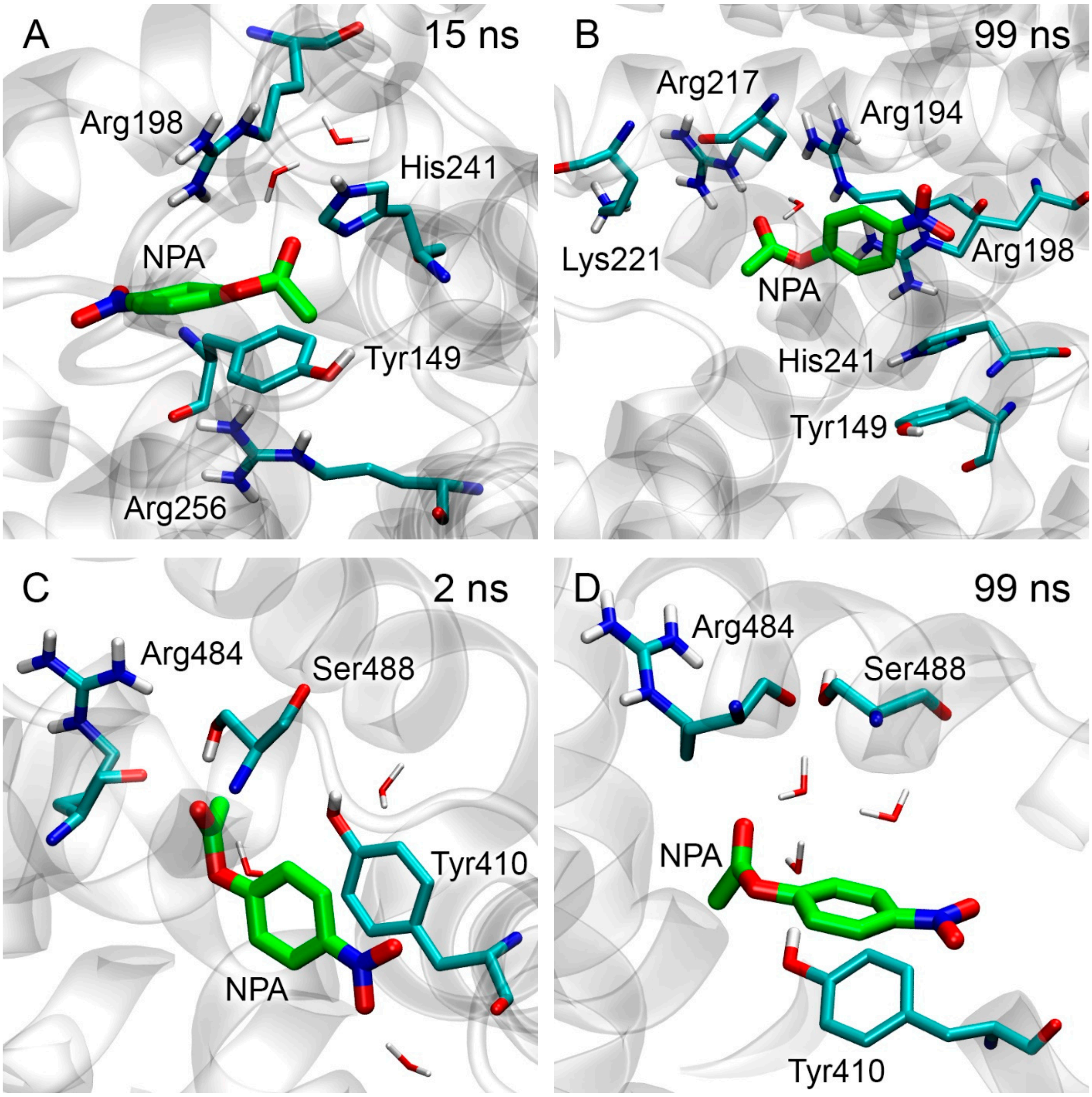

Figure S4. Interaction of NPA with sites Sudlow I and II of free BSA according to MD simulation. A – position of NPA in site Sudlow I after 15 ns of the simulation. B – position of NPA in site Sudlow I after 99 ns of the simulation. C – position of NPA in site Sudlow II after 2 ns of the simulation. D – position of NPA in site Sudlow II after 99 ns of the simulation.
